# Role of Structural and Compositional Changes of Cu_2_O Nanocubes in Nitrate Electroreduction to Ammonia

**DOI:** 10.1021/acsaem.4c02326

**Published:** 2024-10-02

**Authors:** Igor Messias, Manuel E. G. Winkler, Gabriel F. Costa, Thiago Mariano, João Batista Souza Junior, Itamar Tomio Neckel, Marta C. Figueiredo, Nirala Singh, Raphael Nagao

**Affiliations:** † Institute of Chemistry, University of Campinas, Campinas, SP 13083-862, Brazil; ‡ Center for Innovation on New Energies, University of Campinas, Campinas, SP 13083-084, Brazil; § Department of Chemical Engineering, University of Michigan, Ann Arbor, Michigan 48109-2136, United States; ∥ Brazilian Nanotechnology National Laboratory (LNNano), Brazilian Center for Research in Energy and Materials, Campinas, SP 13083-100, Brazil; ⊥ Brazilian Synchrotron Light Laboratory (LNLS), Brazilian Center for Research in Energy and Materials, Campinas, SP 13083-100, Brazil; # Department of Chemical Engineering and Chemistry, Eindhoven University of Technology, Eindhoven, MB 5600, The Netherlands; ∇ Eindhoven Institute of Renewable Energy Systems, Eindhoven University of Technology, Eindhoven, MB 5600, The Netherlands

**Keywords:** electrocatalysis, nitrate reduction, Cu_2_O nanocubes, oxide-derived Cu electrocatalyst, *in situ* spectro-electrochemistry

## Abstract

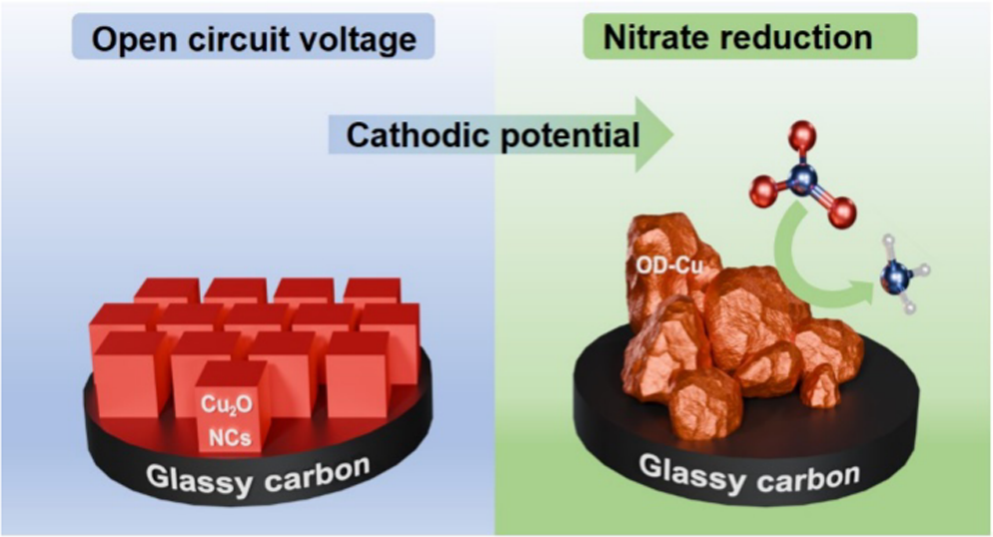

Nitrate electroreduction reaction (NO_3_RR) to ammonia (NH_3_) still faces fundamental and technological challenges. While Cu-based catalysts have been widely explored, their activity and stability relationship are still not fully understood. Here, we systematically monitored the dynamic alterations in the chemical and morphological characteristics of Cu_2_O nanocubes (NCs) during NO_3_RR in an alkaline electrolyte. In 1 h of electrolysis from −0.10 to −0.60 V vs RHE, the electrocatalyst achieved the maximum NH_3_ faradaic efficiency (FE) and yield rate at −0.3 V (94% and 149 μmol h^–1^ cm^–2^, respectively). Similar efficiency could be found at a lower overpotential (−0.20 V vs RHE) in long-term electrolysis. At −0.20 V vs RHE, the catalyst FE increased from 73% in the first 2 h to ∼90% in 10 h of electrolysis. Electron microscopy revealed the loss of the cubic shape with the formation of sintered domains. *In situ* Raman, X-ray diffraction (XRD), and *in situ* Cu K-edge X-ray absorption near-edge spectroscopy (XANES) indicated the reduction of Cu_2_O to oxide-derived Cu^0^ (OD-Cu). Nevertheless, a remaining Cu_2_O phase was noticed after 1 h of electrolysis at −0.3 V vs RHE. This observation indicates that the activity and selectivity of the initially well-defined Cu_2_O NCs are not solely dependent on the initial structure. Instead, it underscores the emergence of an OD-Cu-rich surface, evolving from near-surface to underlying layers over time and playing a crucial role in the reaction pathways. By employing *online* differential electrochemical mass spectrometry (DEMS) and *in situ* Fourier transform infrared spectroscopy (FTIR), we experimentally probed the presence of key intermediates (NO and NH_2_OH) and byproducts of NO_3_RR (N_2_ and N_2_H_*x*_) for NH_3_ formation. These results show a complex relationship between activity and stability of the nanostructured Cu_2_O oxide catalyst for NO_3_RR.

## Introduction

The intensive use of nitrogenous fertilizers has disrupted the nitrogen cycle, as indicated by the accumulation of nitrate in water streams, leading to harmful consequences for both the environment and human health.^1^ The electrochemical nitrate (NO_3_^–^) reduction reaction (NO_3_RR) is acknowledged as a sustainable approach for reducing nitrate levels in contaminated water.^2,3^ Considering the increasing efforts to make the use of electricity from renewable sources viable, electrochemical water remediation is a promising alternative to convert water-contaminants into valuable products, especially ammonia (NH_3_).^4,5^ In addition, the NO_3_RR to NH_3_ is particularly advantageous, considering that ammonia can be used as an energy carrier due to the hydrogen content and also offers a decarbonized path for fertilizer production.^6,7^

The electrochemical conversion of NO_3_^–^ to NH_3_ is a complex multistep process that involves the transfer of eight electrons for both deoxygenation and protonation of the nitrate.^8^ Therefore, the faradaic efficiency (FE) for NH_3_ depends on the relative rates of side reactions that can convert NO_3_^–^ to NO_2_^–^, NO, NH_2_OH, N_2_O, or N_2_.^9^ Additionally, at sufficiently high overpotentials, the hydrogen evolution reaction (HER) also occurs, further diminishing the NO_3_RR FE.^10^ In terms of practical applications, this is a critical limitation due to the HER being favored at the small nitrate concentration range (5–30 ppm) in real polluted sources.^11^ In this scenario, electrocatalyst engineering must consider the adsorption energy and reaction rates of the intermediates involved in these reactions to design materials that improve the selectivity for NH_3_ over the HER.

Copper (Cu) is an earth-abundant catalyst that has high activity and selectivity for NH_3_ production, outperforming other transition metals, such as nickel, cobalt, and iron.^12−14^ Cu electrodes, however, strongly adsorb NO_3_RR intermediates, such as NO_2_^–^ and NO, which causes surface poisoning.^15^ As a result, an overpotential is required to overcome this barrier and fully convert NO_3_^–^ to NH_3_, in which the NO_3_RR and HER are competing reactions. The performance of Cu-electrocatalysts has been improved by alloying Cu with catalytically active elements, such as Ni^12^ and Sn,^16^ and/or incorporating onto substrates (graphitized carbon^17^ and covalent triazine framework^18^).

The oxidation state of the Cu electrocatalyst plays an important role in the efficiency of NH_3_ production. Several studies showcase the effectiveness of employing Cu/Cu-oxides mixed phases to enhance the selectivity toward ammonia.^19−22^ The heightened performance is predominantly ascribed to the diminished energy barrier for the hydrogenation of NO_3_H_ads_ and NO_2_H_ads_ facilitated by Cu^+^ sites. However, the oxidized sites (Cu^2+^ and Cu^+^) are mostly electrochemically reduced to oxide-derived Cu^0^ sites (OD-Cu) during the NO_3_RR, which have been shown to be highly efficient at reducing NO_3_^–^ to ammonia.^15,22^

The relationship of Cu-based materials structure activity/stability toward the NO_3_RR is an important factor for NH_3_ production. For the Cu^0^ surface, previous experimental and theoretical works have demonstrated that the selectivity of ammonia reaction pathways depends on the Cu catalyst crystal orientation.^8,23−25^ Specifically, the most stable atomic-surface arrangement (111) exhibits higher selectivity for ammonia than the (100) surface in neutral and alkaline electrolytes.^8,23^ This observation is associated with the higher free energy of H_2_ than any step for the NO_3_RR.^8^ In terms of kinetics, the improved performance of Cu(111) surfaces is linked to the lower overpotential to convert NO_3_^–^ to NO_2_^–^ than the (100) arrangement.^8^ In contrast, certain reports attribute the higher ammonia selectivity to greater exposure of the Cu(100) facets and thus the higher rate to convert NO_2_^–^ to NH_2_OH than the Cu(111).^23,24,26^ Considering these statements, the performance of preferentially oriented OD-Cu catalysts cannot be assigned to the pristine catalytic surface, but to the structural and morphological changes induced by the cathodic potential.^15,27−29^ Instead, it must be closely tied to the reconstructed surface, which can lead to a misinterpretation of the reaction pathways. Consequently, there is a growing interest in comprehending the dynamic evolution of the Cu structure under operational conditions.^15,28,30,31^ Therefore, it is crucial to unveil the relationship between activity and stability to design surfaces that demonstrate improved performance while maintaining stability for the electrochemical reduction of nitrate to ammonia.

Herein, we provide a systematic experimental approach to understand the structural and compositional evolution of well-defined Cu_2_O nanocube (NCs) particles during the nitrate electrochemical reduction to ammonia. We synthesized Cu_2_O nanoparticles with cubic shape using a wet-chemical ligand-free method and tested their performance toward NH_3_ production in an alkaline electrolyte. We showed that the catalyst presented a maximum FE to ammonia of 94% at −0.3 V vs RHE in 1 h of electrolysis. With long-term electrolysis (10 h at −0.2 V vs RHE), we observed an increase in FE up to 10 h, reaching 92%. By combining *in situ* and *ex situ* techniques with electrochemical experiments, we evaluate the impact of compositional and morphological changes of Cu_2_O nanocubes on the intermediate’s distribution for ammonia production. We observed that the pristine well-defined cubic morphology is not a determining factor for the high ammonia efficiency but rather the evolution of the catalyst during the NO_3_RR. We also tracked the presence of key reaction intermediates using *in situ* Fourier transform infrared spectroscopy (FTIR) and *online* differential electrochemical mass spectrometry (DEMS) to investigate the reaction mechanism. We found that NH_3_ is mainly formed through NO hydrogenation, which leads to the formation of NH_2_OH, further converting into NH_3_, with N_2_ and N_2_H_*x*_ as byproducts.

## Results and Discussion

### Synthesis and Characterization of Cu_2_O Nanocubes (NCs)

**Figure 1:**
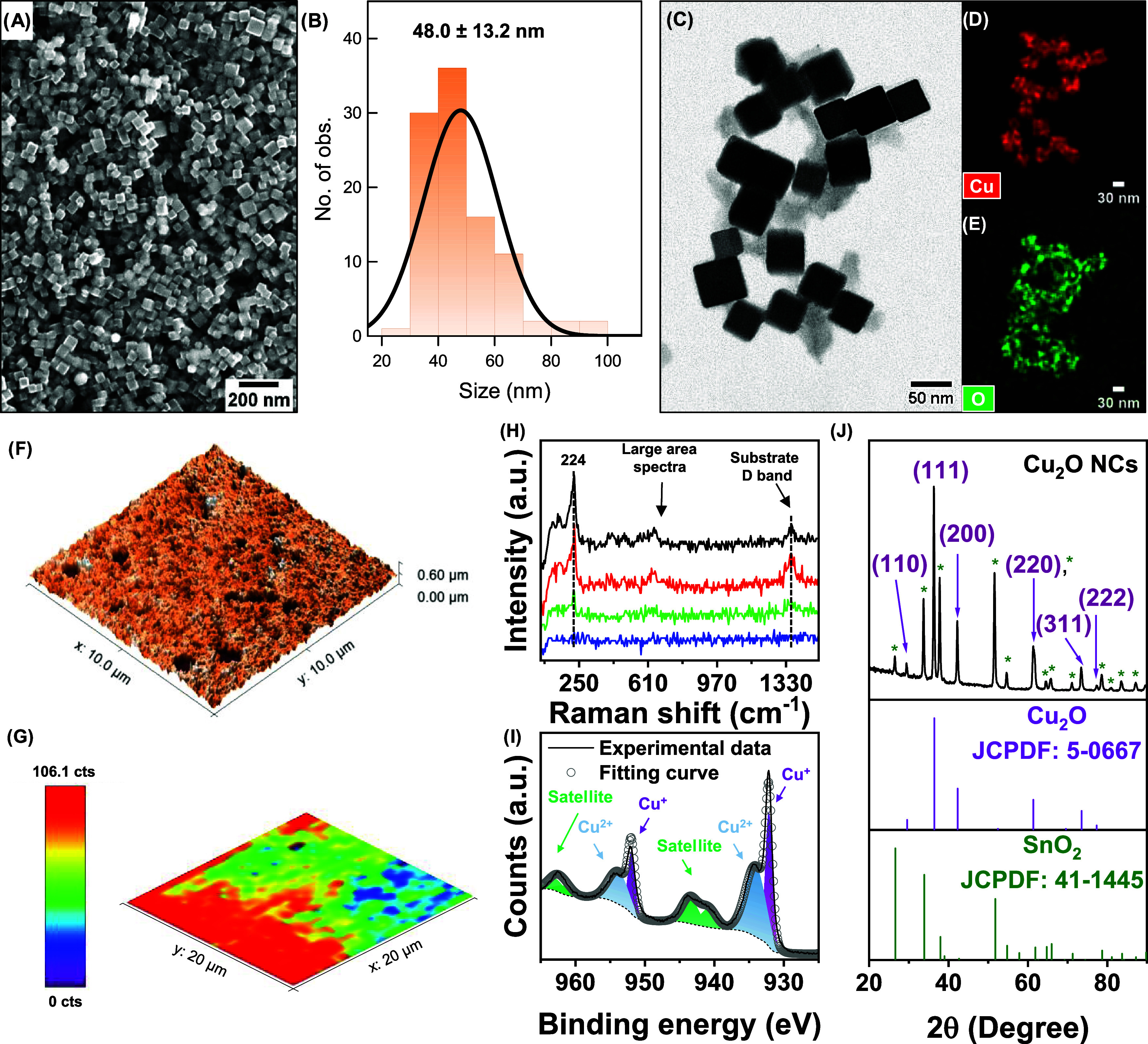
Structural and chemical characterization of as-prepared Cu_2_O NCs. (A) SEM image of the Cu_2_O NCs on the glassy carbon substrate. (B) Particle size histogram acquired from the SEM image shown in Figure S1. Also listed are the mean and standard deviation of particle sizes. (C) BF-STEM image and corresponding EDX maps of (D) Cu and (E) O elements. (F) AFM image capturing a 10 μm × 10 μm section of the Cu_2_O NCs deposited on GC. (G) *Ex situ* Raman mapping of the 224 cm^–1^ signal of Cu_2_O NCs deposited on GC in a 20 μm × 20 μm area. (H) Raman spectra of blue, green, and red regions from panel (G) and the total region analyzed (in black). (I) High-resolution Cu 2p XPS spectra of Cu_2_O NCs. (J) GIXRD pattern of Cu_2_O NCs deposited on the FTO substrate, Cu_2_O, and SnO_2_. Cu_2_O peaks are labeled with the corresponding plane, and SnO_2_ peaks are indicated by stars.

We synthesized Cu_2_O NCs by reducing copper(II) chloride in basic medium using l-ascorbic acid as the reducing agent.^32^ We confirmed the cubic morphology of the particles (Figure 1A) with an average size of 48.0 ± 13.2 nm (Figure 1B) by scanning electron microscopy (SEM); particle size distribution was obtained by measuring the size of 100 particles using ImageJ software (Figure S1). Examining the bright-field scanning transmission electron microscopy (BF-STEM, Figure 1C) image and the corresponding energy dispersive X-ray (EDX) elemental mapping, we verified the homogeneous distribution of Cu and O elements over the catalyst surface (Figure 1D,E). Figure 1F displays the atomic force microscopy (AFM) measurement of a 10 μm × 10 μm size of the catalyst on the glassy carbon (GC) substrate. The micrograph revealed a homogeneous dispersion of the catalyst as a ∼0.60-μm-thick layer. To assess the chemical state of the Cu_2_O NCs and their surface composition, we employed *ex situ* Raman spectroscopy and X-ray photoelectron spectroscopy (XPS). The signal at 224 cm^–1^ is assigned to the characteristic second-order Raman-allowed mode of Cu_2_O,^33^ and Figure 1G shows its intensity distribution over a 20 μm × 20 μm area from a fresh Cu_2_O electrode. The spectra of the colored regions in red, green, and blue are shown in Figure 1H in the same colors; the blue region corresponds to empty spaces where the catalyst is absent, as also observed by SEM (Figure S1). Additionally, the spectrum of the total probed area is represented in black. The characteristic Cu_2_O band (224 cm^–1^)^33^ was observed in the green to red regions and especially in the large-area spectra, as well as the D band of the glassy carbon substrate at 1359 cm^–1^.^34^Figure 1I depicts the high-resolution Cu 2p XPS spectra, revealing two peaks attributed to Cu^+^ (932.5 and 952.1 eV) and Cu^2+^ (at 934.1 and 954.0 eV).^19^ The presence of the Cu^2+^ oxidation state on the catalyst’s surface can be related to unreacted precursor species (Cu(OH)_2_ and Cu(OH)_4_^2–^)^35^ and the unavoidable air exposure of the sample during transportation to the XPS analysis.^28^ To confirm the crystalline phases of the as-prepared catalyst, we utilized *ex situ* grazing incidence X-ray diffraction (GIXRD) patterns due to the thickness of the sample and to preserve the integrity. The X-ray diffractogram (Figure 1J) shows characteristic peaks centered at 2θ = 29.4, 36.3, 42.2, 61.3, 73.4, and 77.4°, attributed to the (110), (111), (200), (220), (311), and (222) planes of cubic Cu_2_O (JCPDF 5-667), respectively. The remaining peaks in the spectra were attributed to the FTO substrate based on the SnO_2_ crystal structure pattern (JCPDF 41-1445).

### Electrochemical Performance for the NO_3_RR

**Figure 2:**
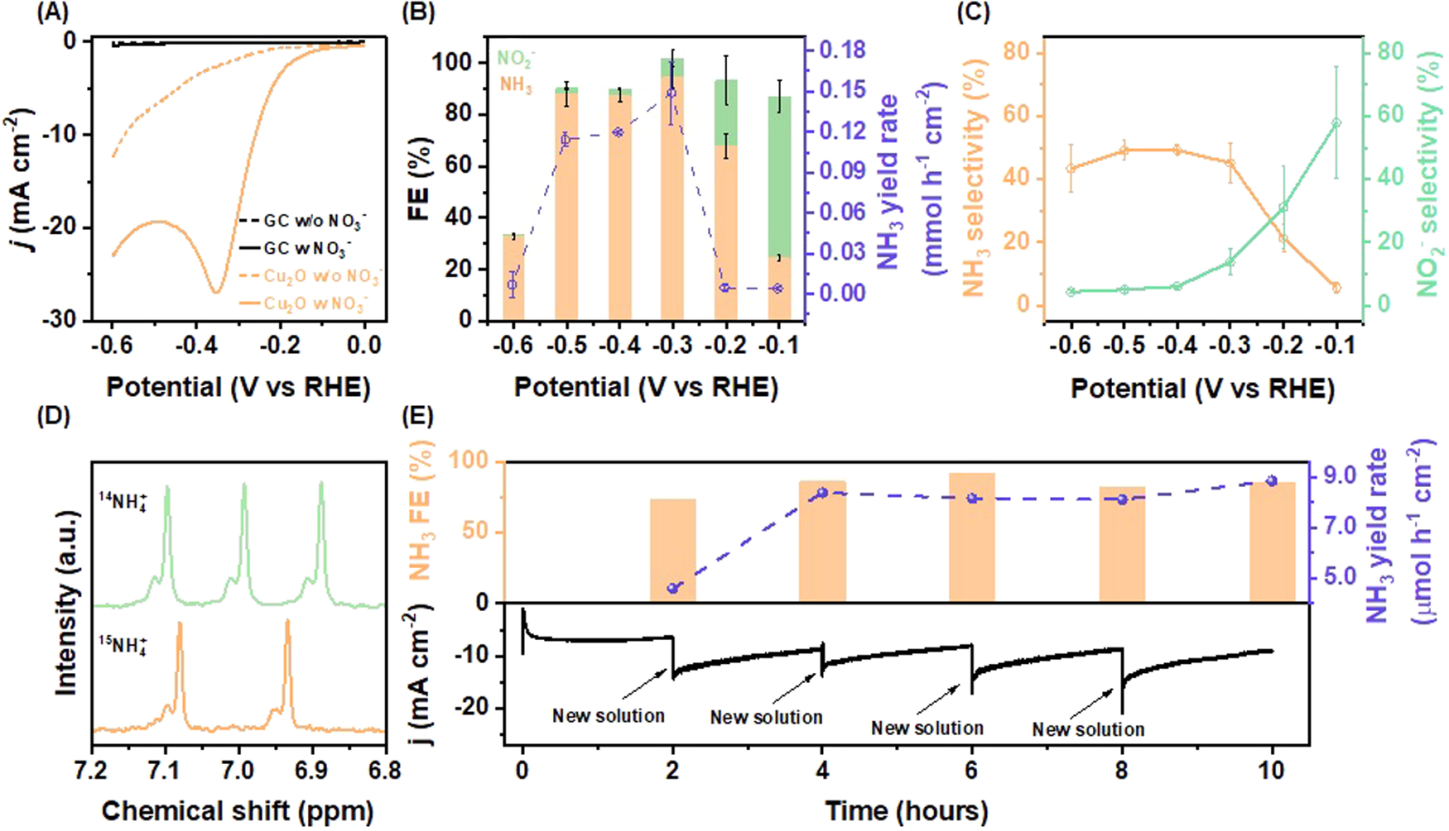
NO_3_RR electrochemical performance of Cu_2_O NCs. (A) LSV curves of the GC substrate (black) and Cu_2_O drop-cast on GC (orange) in 1.0 mol L^–1^ NaOH (dotted line) and 1.0 mol L^–1^ NaOH + 14 mmol L^–1^ NaNO_3_ (solid line). Scan rate: 50 mv s^–1^. Currents were normalized by ECSA. (B) Potential-dependent NH_3_ yield rate (blue line) and FE of the NO_3_RR to NO_2_^–^ and NH_3_ of Cu_2_O NCs in green and orange bars. (C) Selectivity of NO_2_^–^ and NH_3_ at different potentials. (D) ^1^H nuclear magnetic resonance (NMR) spectra of the electrolyte after the NO_3_RR over Cu_2_O NCs at −0.3 V vs RHE used ^14^NaNO_3_ and ^15^NaNO_3_ as the nitrogen source. (E) Chronoamperometric stability test at −0.2 V vs RHE and the corresponding NH_3_ FE and yield rate.

To investigate the electrocatalytic activity of the Cu_2_ O NCs for the NO_3_RR to NH_3_, we conducted linear sweep voltammetry (LSV) and chronoamperometry experiments using a glassy carbon electrode modified with Cu_2_O NCs as the working electrode. Figure 2A shows the LSV curve normalized by the electrochemical active surface area (ECSA) of the electrocatalyst and its substrate (glassy carbon) in 1.0 mol L^–1^ NaOH (dashed orange and black, respectively) and with 14 mmol L^–1^ NaNO_3_ (solid black and orange lines, respectively)—ECSA was determined by measuring the double-layer capacitance (Figure S2) as described in the Supporting Information. The glassy carbon shows negligible current with and without nitrate (Figure 2A) and so the current can be attributed to the Cu_2_O NCs. The Cu_2_O drop-casted on glassy carbon showed an increase in the cathodic current within the negative applied potential range in the presence of nitrate, exhibiting an onset potential at 0.1 V vs RHE. As the applied potential becomes more negative, the increase becomes more pronounced, potentially attributed to the NO_3_RR and HER. Furthermore, in Figure 2A (solid orange line), a peak emerges between −0.3 and −0.4 V vs RHE with a 5-fold higher current density than in the absence of nitrate. The cathodic current density decreases below −0.4 V vs RHE, suggesting a surface site blockage for nitrogen-containing molecules caused by the adsorption of hydrogen.^8,36,37^

To identify the nitrate-reduction products and evaluate the catalyst efficiency, we performed 1 h chronoamperometric electrolysis in a solution containing 1.0 mol L^–1^ NaOH and 14 mmol L^–1^ NaNO_3_ in a potential range of −0.1 to −0.6 V vs RHE in potential increments of 0.1 V. Figure 2B displays the FE values for NH_3_ and NO_2_^–^ (orange and green bars, respectively), and the NH_3_ yield rate (purple line, both products quantified by colorimetric methods)^35^—the analytical curves with excellent goodness factors are shown in Figure S3. At low overpotentials (e.g., −0.1 V vs RHE), we observed the highest FE for NO_2_^–^ and the smaller current density as shown in Figure S4. As the potential is shifted from −0.1 to −0.3 V vs RHE, the FE for NO_2_^–^ decreases from 63 to 8%, while the FE for NH_3_ increases from 24 to 94%. The decrease for NO_2_^–^ with increasing cathodic potential was expected, as NO_2_^–^ is an intermediate for the formation of NH_3_.^23,36^ The FE and yield rate for NH_3_ is the highest at −0.3 V vs RHE (FE: 94 ± 4% and yield rate: 149 μmol h^–1^ cm^–2^) accompanied by a current density 3-fold higher than at −0.1 V. The maximum in FE and yield rate at −0.3 V is consistent with the location of the LSV peak in Figure 2A. We observed a decrease in FE and yield rate toward NH_3_ between −0.4 and −0.6 V vs RHE, indicating a potential domain where the hydrogen adsorption competes for active sites with the NO_3_RR. The potential of the highest FE to NH_3_ (−0.3 V vs RHE) agrees with what was reported in the literature for preferential oriented-Cu_2_O catalyst^28^—76% for Cu_2_O (111) and 68% for Cu_2_O (100)—and is significantly lower than the Cu/Cu_2_O nanowires^19^ (−0.85 V vs RHE). Detailed comparison of the Cu_2_O NC performance with electrocatalysts found in the literature is presented in Table S1. Another important parameter for evaluating the performance of an electrocatalyst is its selectivity, especially for ammonia production.^9^ In this case, we calculated the selectivity for NH_3_ and NO_2_^–^ at the same potential range, which is presented in Figure 2C. Nitrite selectivity decreases rapidly with increasing potential to negative values, while for NH_3_ it reaches a plateau at −0.3 V vs RHE and remains almost constant up to −0.6 V vs RHE, which means that from −0.3 V vs RHE, increasing the overpotential does not improve the catalyst selectivity toward ammonia. We show that nitrate is the nitrogen source for ammonia electrosynthesis through isotopic labeling, followed by product identification by ^1^H NMR. By using ^14^NaNO_3_ in the electrolyte, we found three characteristic symmetrical ^1^H signals of ^14^NH_4_^+^, while with ^15^NaNO_3_ only two symmetrical ^1^H signals of ^15^NH_4_^+^ are observed (Figure 2D). These results suggest that NO_3_^–^ is the main source of nitrogen for ammonia formation.

By testing the catalyst activity and selectivity over time, we observed modification of the catalyst under operating conditions that resulted in an FE increase to ammonia. We carried out five consecutive 2 h of electrolysis at −0.2 V vs RHE, renewing the electrolyte every 2 h, and the results are shown in Figure 2E. We found that the FE and yield rate to ammonia increased over time: FE started at 73%, reached the maximum at 92% after 6 h, and kept around ∼90% until the end of the experiment; and the yield rate increased from 4.6 μmol h^–1^ cm^–2^ in the first 2 h and remained between 8.2 and 8.9 μmol h^–1^ cm^–2^ until the end of the electrolysis. The gradual decrease in current density during the stability test can be attributed to the ongoing reduction of the material to the Cu^0^ phase. This transition alters the surface characteristics of the material, ultimately enhancing its catalytic activity for ammonia production. Notably, the faradaic efficiency (FE) increases after the first 6 h, indicating that the material continues to evolve even after the initial phase transformation observed at 1 h. This prolonged change suggests that the material is still undergoing significant modification, which stabilizes over time, leading to an improved performance. Möller et al. similarly observed a decrease in current density after 3 h of electrolysis at a constant potential on Cu_2_O nanocubes during CO_2_ reduction.^38^ By monitoring the compositional and structural changes, they identified the formation of irregular Cu aggregates, consistent with the observations in our study. Regarding nitrate electroreduction on this type of material, Costa et al. demonstrated that the Cu metallic phase, derived from a surface initially composed of a Cu_2_O/Cu mixture, exhibits intrinsic activity for nitrate electroreduction at certain potentials. The reduction of the oxide layer leads to the formation of defects on the catalyst surface, which likely contribute to its catalytic performance.^39^ To investigate how the electrolysis impacts the structure and composition of Cu_2_O NCs under the NO_3_RR, the next section will detail characterizations performed after the electrochemical reaction.

### Post-electrolysis and *In Situ* Electrocatalyst Surface Characterization

**Figure 3:**
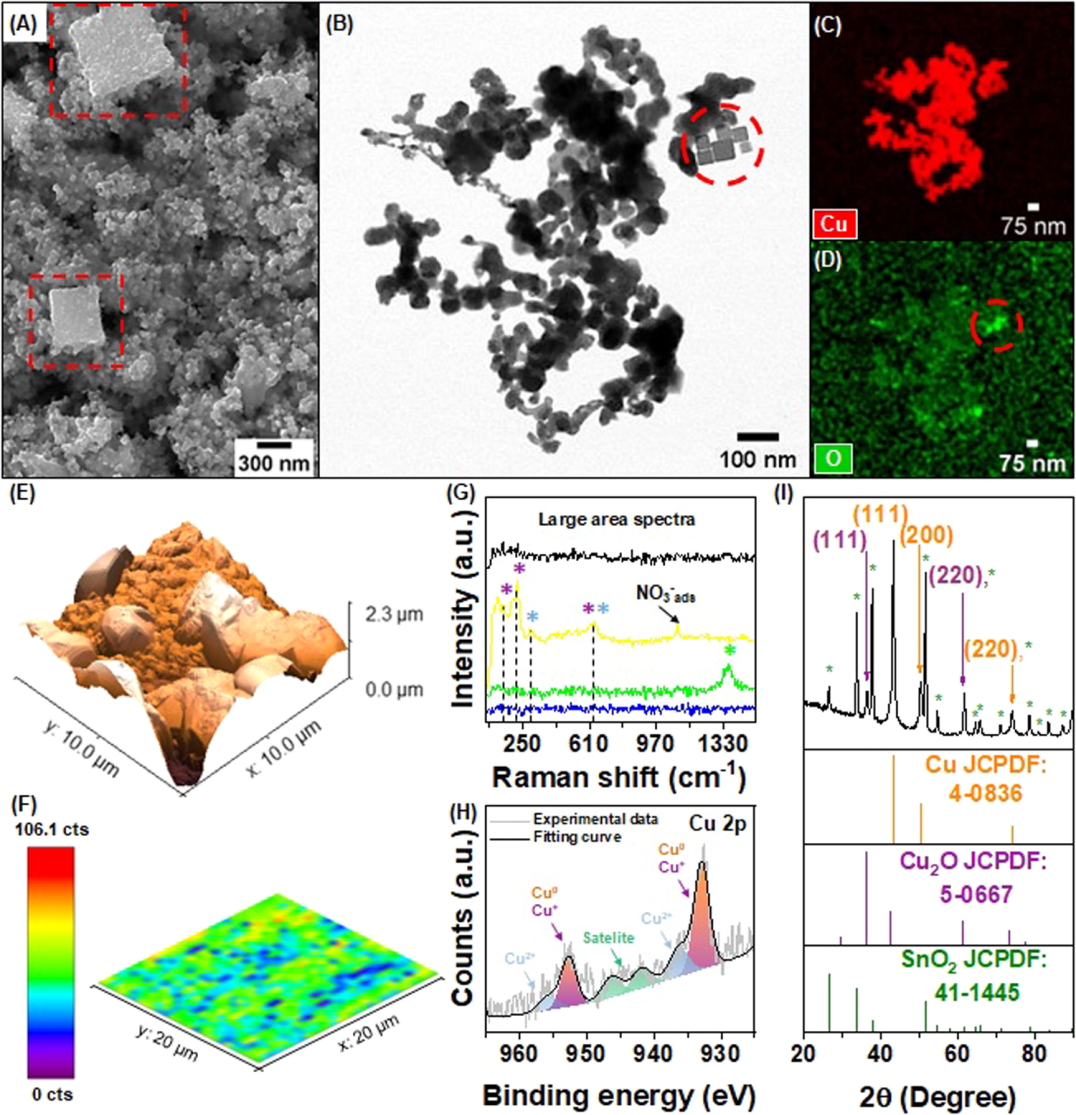
Structural and chemical characterization of the postelectrolysis Cu_2_O NCs in 1.0 mol L^–1^ NaOH + 14 mmol L^–1^ NaNO_3_ at −0.3 V vs RHE after 1 h. (A) SEM image of the Cu_2_O NCs on the glassy carbon substrate. (B) BF-STEM image and corresponding EDX maps of (C) Cu and (D) O elements. (E) AFM image capturing a 10 μm × 10 μm section of the Cu_2_O NCs deposited on GC. (F) *Ex situ* Raman mapping of the 224 cm^–1^ signal of Cu_2_O NCs on GC in a 20 μm × 20 μm area and (G) corresponding-colored spectra of the regions in panel (F) and the entire region analyzed. (H) High-resolution Cu 2p XPS spectra. (I) GIXRD pattern of Cu_2_O NCs deposited on the FTO substrate. Cu, Cu_2_O, and SnO_2_ standards are shown at the bottom.

We observed substantial morphological and structural evolution of the Cu_2_O NCs during the NO_3_RR. Figure 3A,B displays the SEM and BF-STEM images of the catalyst after 1 h of electrolysis at −0.3 V vs RHE in 1.0 mol L^–1^ NaOH and 14 mmol L^–1^ NaNO_3_. After this period, the surface of Cu_2_O NCs presented shape deformation accompanied by a sintering process. In contrast to the as-prepared sample, the smaller particles (∼50 nm) loose the initial shape, whereas larger particles (average size ranging from 150 to 600 nm) exhibit rougher surfaces and fewer morphological changes (Figures 3A and S5). This illustrates the susceptibility of the oxide surface to instability under cathodic potential, as the initially well-defined particles undergo a near-complete loss of their cubic morphology. Additionally, we observed some preserved nanoparticles in the BF-STEM image (Figure 3B), possibly indicating that an underneath particle layer remains stable after the applied potential. The corresponding elemental mapping shows the distribution of Cu and O (Figure 3C,D). The Cu map displayed a uniform distribution across the sample, as in the as-prepared sample in Figure 1D. However, the intensity of the O map (Figure 3D) was lower than that of the O map acquired before the electrolysis (Figure 1E). Notably, the cubic domains, with preserved morphology, exhibited the highest oxygen intensity region, supporting that the oxide underlayer remains unexposed to the electrolyte during the electrolysis and therefore preserved their chemical composition. Based on this, the increase in FE for NH_3_ over time, as well as the sustained yield rate, would not be correlated with the cubic morphology and pristine chemical composition, but rather with characteristics caused by the reduction of Cu_2_O to Cu^0^, such as oxygen vacancies and stacking faults as proposed in the literature.^15,27−29^

Comparing AFM images of pristine (Figure 1F) and postelectrolysis (Figure 3E) Cu_2_O NCs, we noticed a pronounced increase in the grain size and layer thickness (from 0.6 to 2.3 μm). This may occur by the grain sintering or by dissolution and redeposition of copper, the latter of which has already been shown in the literature for both the NO_3_RR and CO_2_ reduction.^28,40^ ECSA determination before and after electrolysis revealed a 6.5-fold increase with the applied potential, suggesting that even with the increase in grain size, the number of active sites also increased (Figure S6).

The chemical composition of the Cu_2_O NCs also changed during reaction, primarily from the partial reduction of Cu_2_O to Cu^0^, leading to a mixture of Cu^0^ and Cu_2_O. A simple indicator is the shift in the color of the electrode from orange pristine to black in the postelectrolysis (Figure S7). The copper oxide structure and, consequently, the chemical composition was assessed by Raman spectroscopy. We investigated the Raman peak at 224 cm^–1^ attributed to Cu_2_O in a 20 μm × 20 μm area (Figure 3F), which revealed an intensity 2.3-fold lower than that observed in the fresh electrode (Figure 1G). This diminished signal across the analyzed area is attributed to Cu_2_O reduction to Cu^0^ and the nonhomogeneity catalyst distribution (observed by X-ray fluorescence (XRF) mapping and SEM micrographs in Figure S8A,B). However, we observed spots where a mixed oxide phase is evident (colored in yellow in Figure 3F), exhibiting signals at 150 and 225 cm^–1^ associated with Cu_2_O, along with a signal at 285 cm^–1^ ascribed to CuO (Figure 3G). Additionally, there is a signal at 1085 cm^–1^ related to adsorbed nitrate, indicative of the remaining species on the catalyst surface.^33,41^ To evaluate the catalyst’s phases, facet distribution, and copper oxidation states, we additionally conducted XPS and GIXRD measurements of the postelectrolysis electrode (Figure 3H,I, respectively). The Cu 2p XPS results indicated the presence of two sharp peaks centered at 932.5 eV (Cu 2p_3/2_) and 952.4 eV (Cu 2p_1/2_), both attributed to the Cu^+^/Cu^0^ oxidation state on the catalyst near-surface due to the Cu LMM peaks at 918.6 and 917 eV (Figure S9).^42,43^ Moreover, a residual presence of Cu^2+^ (broad peak at 934.1 eV) is observed on the catalyst surface, likely resulting from surface oxidation by the alkaline electrolyte and air exposure.^28^ To support this hypothesis, two Cu_2_O NC electrocatalysts were submitted to electrolysis at identical electrochemical parameters (1 h at −0.3 V vs RHE in Ar-saturated NaOH 1.0 mol L^–1^ and NaNO_3_ 14 mmol L^–1^) followed by XPS characterization. The increased CuO content (Figure S10) was attributed to the unavoidable exposure of the postelectrolysis electrodes due to the same electrochemical procedure. The contribution of the Cu^0^/Cu^+^ peak corresponded to 76%, while Cu^2+^ accounted for 24%. The presence of CuO, however, is attributed to unavoidable oxidation upon exposure to atmospheric air.^32^ This is supported by *in situ* Raman measurements (Figure S11), revealing two pronounced peaks at 295 and 340 cm^–1^ attributed to CuO species,^33^ the presence of these oxides persists throughout the entire evaluated potential range, both in the absence of nitrate and during the NO_3_RR (Figure S9A,B). Interestingly, we detected three additional peaks centered at 150, 520, and 620 cm^–1^ related to Cu_2_O species, evidencing the presence of an oxidized copper phase on the catalyst surface.^33^ Furthermore, the GIXRD pattern evidenced the presence of polycrystalline Cu^0^ with the crystallographic planes (111) at 2θ = 43.26°, (200) at 2θ = 50.35°, and (220) at 2θ = 74.21°, confirming the reduction of Cu_2_O postelectrolysis. However, we also identified a residual presence of Cu_2_O with the planes (111) and (220) at 2θ = 36.32 and 61.72°, respectively, suggesting that the catalyst may possess a combination of Cu^+^/Cu^0^ phases. Nevertheless, *ex situ* XRD analysis may not provide the most accurate results for characterizing the postelectrolysis surface, primarily due to the inherent instability of Cu when exposed to air. Therefore, we conducted *in situ* Cu K-edge X-ray absorption near-edge spectroscopy (XANES) to investigate the Cu*^x^* active site for the NO_3_RR. The *in situ* XANES spectra were collected at OCP, −0.2, −0.4, and −0.6 V vs RHE (Figure S12A). To estimate *E*_0_ at each applied potential, the maxima of the first derivative curves were determined (Figure S12B). We observed the Cu K-edge peak at 8983 eV at open-circuit voltage (OCP), attributed to Cu_2_O,^44^ and as the cathodic potential was applied, this peak shifted toward the Cu^0^ value at ∼8980 eV^39^ (Figure S12C). These results are consistent with the reduction of Cu_2_O to Cu^0^ during the NO_3_RR. We also hypothesized that the presence of the remaining Cu_2_O species is related to possible: (i) loss of electric contact due to a weak binding of some NCs with the substrate or (ii) by the presence of an underneath oxide particle layer that is not exposed to the electrolyte, even after 1 h applying a negative potential (−0.3 V vs RHE).

Based on the foregoing results, we propose that the performance of the oxide-derived Cu surface is associated with the Cu^0^ surface, instead of the Cu_2_O/Cu^0^ sites. The observed correlation is influenced by the variations in the probing depth among the utilized techniques. Thus, under an applied potential, the process initiates with a rapid surface reduction, inducing shape loss, and gradually extends to underneath layers over time. This phenomenon may account for the gradual increase in FE observed during 10 h of electrolysis. It suggests that, following the initial 6 h, the electrocatalyst achieves a Cu^0^ composition that exhibits higher activity than the mixture of oxide and metallic phases, demonstrating that the 10 h duration was sufficient to capture the material’s dynamic evolution. Möller et al.^38^ observed the same surface behavior during CO_2_ electroreduction to C_2+_ products on Cu_2_O NCs. The changes of the catalyst’s structure and composition toward a Cu^0^-rich surface were identified as a pivotal factor contributing to sustained catalytic enhancement, demonstrated over a continuous period of 40 h. They conclude that the emergence of defects in the Cu lattice during the reduction of the oxide contributes to the observed efficiency. Moreover, for the NO_3_RR, the Cu^0^ surface derived from Cu_2_O has also been described as the active phase. The electrochemical reduction of the oxide creates Cu^0^ sites more active and selective to ammonia than the polished Cu^0^ surface.^22,28,29^ Roldan Cuenya et al.^42^ showed that the electrocatalytic performance for NO_2_^–^ at low potentials is associated with Cu_2_O sites, and for NH_3_ with Cu^0^ sites at higher potentials. Similarly, our findings show that the reconstructed Cu_2_O NCs, which may generate Cu uncoordinated atoms, are the NO_3_RR active sites.

In light of these findings and with data from the literature,^25^ corroborating that the initial structure of Cu_2_O—such as cubic—does not play a pivotal role in the performance of the catalyst, a natural question that raises is what advantages cubic-shaped Cu_2_O catalysts have when compared to a preoxidized bulk Cu electrode? Preoxidized bulk Cu has a smoother and less defective surface, exhibiting lower faradaic efficiency for ammonia due to fewer active sites and a less favorable surface for nitrate adsorption and reduction.^24^ Defect-rich surfaces, such as those found in OD-Cu, can improve the adsorption of nitrate and intermediates, thus enhancing the overall catalytic performance.^24,25,28,39^ Therefore, cubic structures maintain advantages over preoxidized bulk Cu as pristine electrocatalyst due to their propensity to form and sustain active sites after structural transformations.

### Role of Cu_2_O NCs in the Reaction Mechanism

The role of these Cu_2_O-based materials in the reaction mechanism of the NO_3_RR is ascribed to different factors. Wang and co-workers^19^ attributed the Cu_2_O/Cu interface as the active site of their catalyst as it promotes the formation of the intermediate NOH_ads_, which is further hydrogenated to produce hydroxylamine (NH_2_OH). Fu et al. showed that the Cu_2_O/Cu interface changes the mobility of the key intermediate and electron transfer, enhancing the NO_3_RR activity.^45^ In addition, Shi and co-workers demonstrated that the presence of oxygen vacancies and the electron transfer from Cu_2_O to Cu in Cu/Cu_2_O nanorods are important for superior catalyst activity. First, Cu vacancy sites promote the reduction of NO_3_^–^ to NO_2_^–^ that are further reduced to NH_3_ due to the presence of adsorbed hydrogen atoms on the Cu_2_O interface.^27^ In an opposing way, Daiyan et al.^46^ found that oxygen vacancies formed in the Cu_2_O lattice favor the formation of the HNO_2,ads_ intermediate, which is further reduced to NO_ads_, HNO_ads_, and H_2_NO_ads_, the last one being responsible for promoting the formation of NH_3_ through a proton–electron pair transfer. Costa et al.^39^ also evaluated by applying *in situ* and *ex situ* techniques that the presence of oxygen vacancies on the Cu oxide-derived surface promotes the NO_3_RR, through the NH_2_OH intermediate formation. In this study, we aimed to determine the role of Cu_2_O-based electrocatalysts in the NO_3_RR with *in situ* FTIR and *online* DEMS.

**Figure 4:**
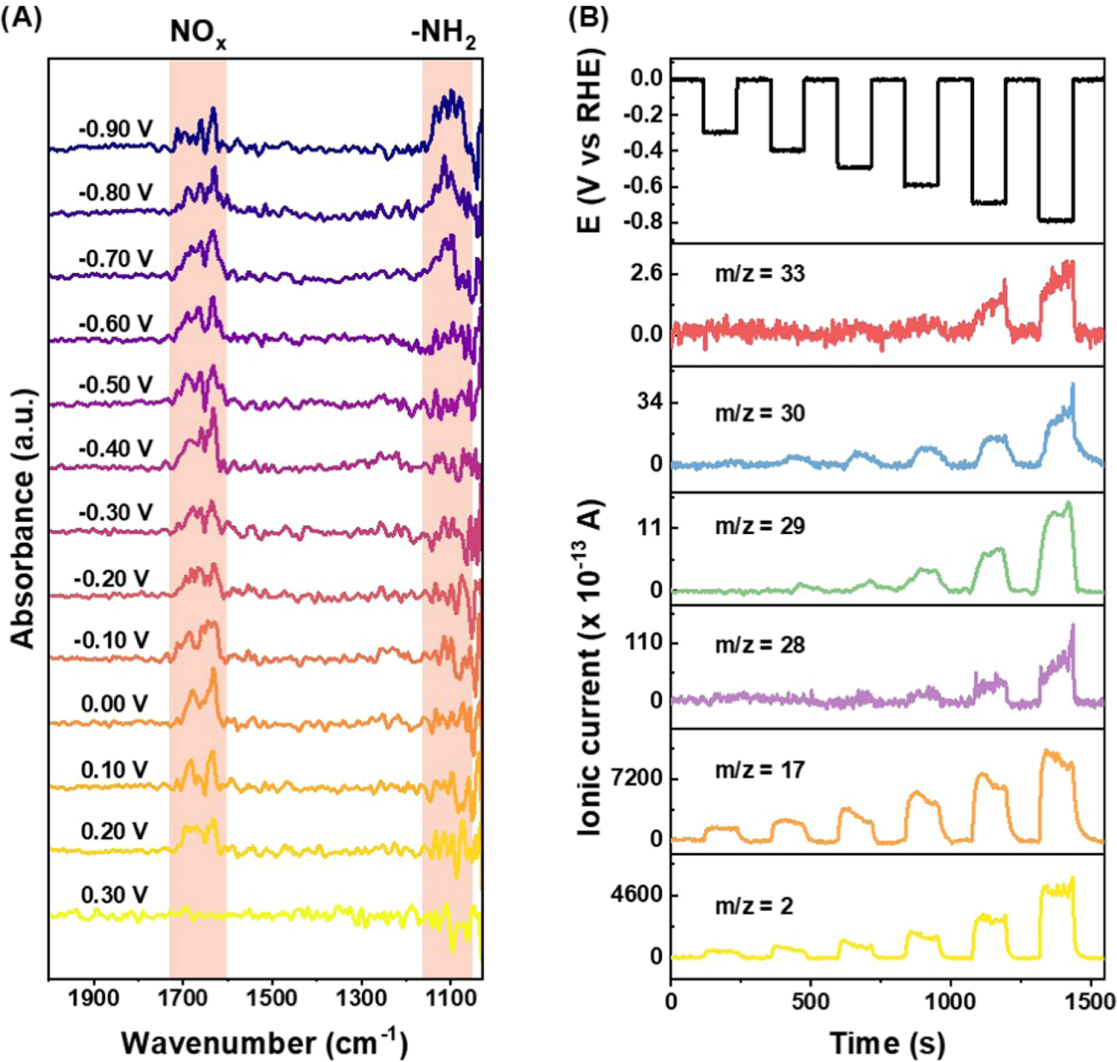
(A) *In situ* FTIR spectra of Cu_2_O NCs in 1.0 mol L^–1^ NaOH electrolyte containing 20 mmol L^–1^ NaNO_3_. The peak centered at 1190 cm^–1^ is related to −NH_2_ from NH_2_OH and the peak centered at 1690 cm^–1^ is related to NO_*x*_ adsorbed species, both highlighted in orange. (B) *Online* DEMS of Cu_2_O NCs recorded in 1.0 mol L^–1^ NaOH + 140 mmol L^–1^ NaNO_3_. The applied potential vs time is represented at the top, followed by the ionic current of possible ionic fragments present in the reaction. The *m*/*z* = 2 corresponds to the H_2_ fragment, while *m*/*z* = 17 represents ionic fragments from NH_3_ and H_2_O. Additionally, *m*/*z* = 28 (N_2_), 29 (N_2_H_2_ or/and N_2_H_4_), 30 (N_2_H_*x*_ or/and NO), and 33 (NH_2_OH) correspond to ionic fragments of possible intermediates of the NO_3_RR.

**Figure 5:**
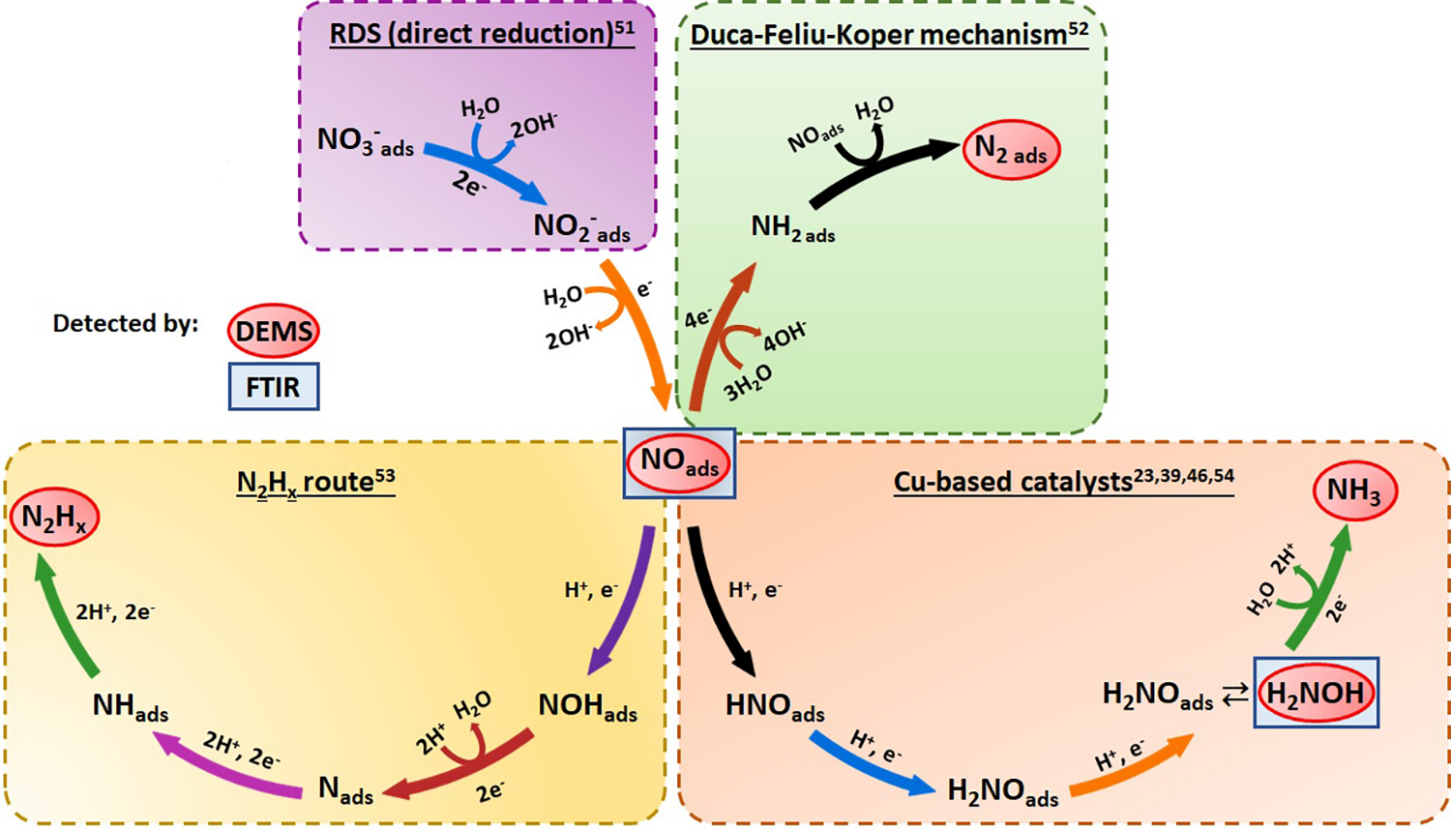
Proposed mechanism for the NO_3_RR by Cu_2_O NC electrocatalysts based on the intermediates identified by *online* DEMS, *in situ* FTIR, and literature data.

We carried out *in situ* FTIR chronoamperometry experiments to identify and monitor the N-content species adsorbed on the catalyst surface, especially NO_*x*_ and NH_2_OH, during the NO_3_RR. We show in Figure 4A an overlapped absorption band between 1610 and 1690 cm^–1^ that increases at 0.2 V vs RHE, ascribed to the O–H bending mode of water molecules and/or the N–O stretching of adsorbed NO_*x*_ intermediates.^2,9,47−49^ Meanwhile, the absorption band around 1190 and 1050 cm^–1^ is observed in the potential range of −0.6 to −0.9 V vs RHE. This is attributed to the –NH_2_ stretch related to hydroxylamine formation.^23,46,48^ The absence of the characteristic nitrite peak at 1231 cm^–1^ indicates the rapid conversion of NO_2_^–^ to NO and, consequently from NO to NH_3_, since nitrite conversion is the rate-determining step in the reaction.^2,50^ For Cu single crystals (111) and (100), this was only observed in acidic media.^23^ The absence of the hydroxylamine-related band was up to −0.6 V vs RHE, suggesting that hydroxylamine is formed at a very low concentration up to this potential, and from this onward, it was detected at all potentials. We also probed the formation of key volatile species by tracking the ionic currents related to different mass/charge ratios (*m*/*z*) with DEMS (Figure 4B). As expected, we detected an increase in the ionic current of *m*/*z* = 2 from −0.3 to −0.8 V vs RHE was assigned to H_2_ formation. This confirms that part of the cathodic current observed in the LSV curve in the presence of nitrate (Figure 2A solid orange line) is associated with the HER. The same increase was observed for *m*/*z* = 17, which could be related to the formation of NH_3_.^50^ However, considering that the same trend was observed for *m*/*z* = 18 (Figure S13), we attribute these signal fluctuations to overlapping of the ammonia and water fragments. The *m*/*z* = 28 corresponds to N_2_, and the increase in the ionic current is first detected from −0.6 V vs RHE, explaining the decrease in the NH_3_ FE (Figure 2B). Additionally, an ionic current of *m*/*z* = 29 was first detected at −0.4 V vs RHE and can be related to the formation of both N_2_H_2_ and N_2_H_4_ (N_2_H^+^ fragment with *m*/*z* = 29). These species are further converted to N_2_, which is better discussed below. The ionic current of *m*/*z* = 30 also increases from −0.4 V vs RHE, being related to both N_2_H_*x*_ species and NO, was also detected by *in situ* FTIR (Figure 4A). We also probed *m*/*z* = 33 related to NH_2_OH, which was first detected from −0.6 V vs RHE, in accordance with *in situ* FTIR as well. This result suggests that between −0.3 to −0.5 V vs RHE, NH_2_OH is rapidly converted to NH_3_, making it untraceable. Thus, in these conditions, NH_2_OH is considered the key intermediate in alkaline electrolytes, as well as NO_*x*_ species.^50^ These observations align with Daiyan and co-workers^46^ who focused on the role of Cu_2_O-based electrocatalysts in the NO_3_RR on sequential hydrogenation of the NO_ads_ intermediate. From the above results, we propose a NO_3_RR mechanism based on the identification of key products and intermediates by *online* DEMS and *in situ* FTIR results (Figure 5). In the first step, NO_3_^-^_ads_ is converted to NO_2_^-^_ads_ by a two-electron transfer reaction, known as the rate-determining step.^51^ NO_2_^-^_ads_ is then reduced to NO_ads_, which is a key intermediate that leads to a series of reaction routes.^50^ From the hydrogenation of NO_ads_, we identified three possible reaction routes for the formation of the products revealed by *online* DEMS and *in situ* FTIR: (i) the N_2 ads_ route via the Duca–Feliu–Koper mechanism,^52^ (ii) the route for the formation of N_2_H_*x*_ species^100^ (N_2_H_2_ or N_2_H_4_); and (iii) the production of ammonia through the reduction of NH_2_OH as the intermediate.^23,39,46,54^ The N_2_H_*x*_ species was identified by Yao and collaborators on rhodium surfaces, who attributed these species as intermediates for NH_3_.^53^ To test this hypothesis, we carried out hydrazine (N_2_H_4_) electroreduction experiments and performed the indophenol test in the electrolyte after 1 h at −0.3, −0.6, and −0.9 V vs RHE to identify and quantify ammonia. However, ammonia was not detected even at more negative potentials (Figure S14). Therefore, we attributed it to byproducts and not to intermediates for NH_3_.

## Conclusions

This study identifies and correlates chemical and structural factors that contribute to the NO_3_RR. Using *ex situ* and *in situ* characterizations, we demonstrate that during the NO_3_RR, a well-defined surface of Cu_2_O NCs is rapidly converted into an oxide-derived Cu^0^ on the near-surface, followed by a gradual reduction of the underneath layers. This surface reconstruction leads to the generation of defects in the Cu lattice acting as the main active sites for the reaction. Despite the structural and compositional changes, the catalyst presented at lower overpotential (−0.2 V vs RHE) an increase in FE from 73 to 92% after the initial 6 h, maintaining this performance for 10 h, highlighting that the active site is not the pristine oxide surface. Furthermore, we established an experimental mechanism investigation to attribute the role of Cu_2_O NC derivate surface in the NO_3_RR to ammonia, in which we found that their main contribution to NH_3_ formation lies in facilitating NO hydrogenation to form NH_2_OH. These findings underscore the paramount significance of comprehending the structural and compositional evolution of the catalyst during the NO_3_RR. This insight provides an indispensable basis for discerning trends in the activity/stability of this reaction on Cu-based catalysts, establishing a basis for the systematic optimization of electrocatalysts with enhanced efficiency.

## Supporting Information Available

Cu_2_O NCs synthesis, experimental details, methods, comparison of catalytic performance of Cu_2_O NCs with others reported in the literature, additional physical characterization: SEM micrographs, XRF map, *in situ* Raman and XANES spectra, *online* DEMS of the *m*/*z* = 18 fragment, and N_2_H_4_ to NH_3_ electroreduction experiments (PDF)

## Abbreviations

AFM: atomic force microscopy
BF-STEM: bright-field scanning transmission microscopy
DEMS: differential electrochemical mass spectrometry
ECSA: electrochemical surface area
EDX: energy dispersive X-ray
FE: faradaic efficiency
FTIR: Fourier transform infrared spectroscopy
GC: glassy carbon
GIXRD: grazing incidence X-ray diffraction
HER: hydrogen evolution reaction
LSV: linear sweep voltammetry
NCs: nanocubes
NMR: nuclear magnetic resonance
NO_3_RR: nitrate-reduction reaction
OD-Cu: oxide-derived copper
SEM: scanning electron microscopy
XANES: X-ray absorption near-edge spectroscopy
XPS: X-ray photoelectron spectroscopy
XRF: X-ray fluorescence
