# Supplementary material for: Role of Structural and Compositional Changes of Cu2O Nanocubes in Nitrate Electroreduction to Ammonia
Source: ACS Appl Energy Mater. 2024 Oct 2;7(19):9034–44. doi: 10.1021/acsaem.4c02326 (PMC11480975; doi:10.1021/acsaem.4c02326)
Supplement: Supplementary file 1 — ae4c02326_si_001.pdf [file ae4c02326_si_001.pdf]

Supporting Information

The Role of Structural and Compositional Changes of Cu_2O Nanocubes in Nitrate Electroreduction to Ammonia

Igor Messias[a$], Manuel E. G. Winkler[a,b$],‡ Gabriel F. Costa[a,c], Thiago Mariano[a,b], João Batista Souza Junior[a,d], Itamar Tomio Neckel[e], Marta C. Figueiredo[f,g], Nirala Singh[c], Raphael Nagao[a,b]*

[a]Institute of Chemistry, University of Campinas, Campinas, SP 13083-862, Brazil
[b]Center for Innovation on New Energies, University of Campinas, Campinas, SP 13083-084, Brazil
[c]Department of Chemical Engineering, University of Michigan, Ann Arbor, MI 48109-2136, USA
[d]Brazilian Nanotechnology National Laboratory (LNNano), Brazilian Center for Research in Energy and Materials, Campinas, SP 13083-100, Brazil;
[e]Brazilian Synchrotron Light Laboratory (LNLS), Brazilian Center for Research in Energy and Materials, Campinas, SP 13083-100, Brazil
[f]Department of Chemical Engineering and Chemistry, Eindhoven University of Technology, Eindhoven, MB 5600, The Netherlands
[g]Eindhoven Institute of Renewable Energy Systems, Eindhoven University of Technology, Eindhoven, MB 5600, The Netherlands

*corresponding author: nagao@unicamp.br

[$]These authors contributed to the manuscript equally

LIST OF FIGURES

Figure S1. SEM image used for particle size determination. S9

Figure S2. Electrochemically active surface area (ECSA) measurements. (A) Cyclic voltammetries of Cu_2O NCs at different scan rates (20 to 100 mV s^{-1}) in 1.0 mol L^{-1} NaOH electrolyte. (B) linear relationship (black line) for the modulus of the average of double-layer current and the scan rate. S10

Figure S3. UV-vis spectra of A) NH_3 and (C) NO_2^- quantifications. Linear relationship between the maximum absorbance wavelength and species concentration for B) NH_3 at 657 nm and D) NO_2^- at 540 nm. S11

Figure S4. Chronoamperometry curves of Cu_2O NCs at different applied potentials from −0.1 V to −0.6 V *vs*. RHE in 1.0 mol L^{-1} NaOH with 14 mmol L^{-1} of NO_3^- under magnetic stirring at 400 rpm. S12

Figure S5. SEM images of the electrocatalyst A) before and B-C) after 1-hour of NO_3RR at −0.3 V *vs*. RHE in 1.0 mol L^{-1} NaOH with 14 mmol L^{-1} of $NaNO_3$ at medium and high magnification, respectively. The red arrows indicate larger nanocubes particles domains. S14

Figure S6. Electrochemically active surface area (ECSA) measurements of Cu_2O electrode before and after 1-h electrolysis (-0.3 V *vs.* RHE in 1.0 mol L^{-1} NaOH and 14 mmol L^{-1} $NaNO_3$). Cyclic voltammetries of Cu_2O NCs and linear relationship (black line) for the modulus of the average of double-layer current and the scan rate A-B) before applying cathodic potential and C-D) after. Cyclic voltammetries were obtained in 1.0 mol L^{-1} NaOH. S15

Figure S7. Photographs of the Cu_2O NCs modified glassy carbon electrode A) before and B) after 1-hour chronoamperometry at −0.3 V *vs*. RHE in NaOH 1.0 mol L^{-1} and $NaNO_3$ 14 mmol L^{-1}. S16

Figure S8. Post-electrolysis characterization of Cu_2O NCs by A) XRF mapping of a 50 μm x 50 μm analyzed area (500 nm/pixel; scale bar denotes 10 μm). Redder regions represent higher Cu concentration; and B) low-magnification SEM micrograph. S17

Figure S9. Cu LMM spectra of pristine and post-electrolysis Cu_2O NCs (1-h at –0.3 V *vs.* RHE in NaOH 1.0 mol L^{-1} and $NaNO_3$ 14 mmol L^{-1}). S18

Figure S10. High-resolution Cu 2p XPS spectra of two post-electrolysis Cu_2O NCs (1-h at –0.3 V vs. RHE in 1.0 mol L^{-1} NaOH and 14 mmol L^{-1} $NaNO_3$). A) Experiment 1 and B) 2. .... S19

Figure S11. *In situ* Raman spectra A) without (potential range: 0.8 to -0.4 V *vs*. RHE) and B) with 0.1 mol L^{-1} of $NaNO_3$ (potential range: 0.8 to 0 V *vs*. RHE) in 1.0 mol L^{-1} NaOH

electrolyte. Pink marks highlight the peaks related to Cu_2O (145, 520, 630 cm^{-1}) and green ones highlight peaks related to CuO (295, 340 cm^{-1}). S20

Figure S12. A) Normalized *in situ* XANES spectra of Cu_2O NCs at different applied potentials in 1.0 mol L^{-1} NaOH and 14 mmol L^{-1}, and the spectra of the standards Cu and Cu_2O; B) the corresponding first derivative curves of the *in situ* XANES spectra; and C) dependence of the Cu K-edge energy as a function of the electrochemical potential. S21

Figure S13. Online DEMS of Cu_2O NCs recorded in 0.1 mol L^{-1} NaOH + 14 mmol L^{-1} $NaNO_3$ electrolyte. A) Applied potential *vs*. time. B) Ionic current *vs*. time of the fragment m/z = 18 of H_2O molecules. S22

Figure S14. Figure S1. UV-vis spectrum of ammonia quantification by chronoamperometry at −0.30 V, −0.60 V and −0.90 V *vs*. RHE in 1.0 mol L^{-1} NaOH and 0.20 mol L^{-1} N_2H_4. ....S23

EXPERIMENTAL PROCEDURE

Synthesis of Cu_2O nanocubes (Cu_2O NCs).

The ligand-free Cu_2O nanocubes were prepared following the Herzog, A. *et al.*, 2021 work.[1] First, 5 mL of a $CuCl_2$ solution (0.1 mol L^{-1}) and 15 mL of a NaOH solution (0.2 mol L^{-1}) were diluted in 200 mL of ultrapure and kept under magnetic stirring (500 rpm) for 5 minutes. Then, 10 mL of L-ascorbic acid solution (0.1 mol L^{-1}) was added to the mixture and stirred for 60 minutes. To remove the unreacted chemicals and the OH^- excess the solution was washed and centrifuged three times, including two with an ethanol-water mixture (1:1) and one with pure ethanol. The resulting precipitate was then re-suspended in 10 mL of ethanol, resulting in a Cu_2O loading of 2 mg mL^{-1} and stored in the refrigerator for further use.

Characterization

Particle morphology and chemical composition were determined by high-angle annular dark-field scanning transmission electron microscopy (HAADF-STEM) coupled with energy-dispersive X-ray (EDX) mapping in a FEI Titan Cubed Themis microscope. FEG-SEM micrographs were acquired in a FEI Quanta 250 microscope operating at 20.00 kV. The topography of the samples were examined with a FlexAFM with C3000 controller (Nanosurf, Switzerland) operating under dry atmosphere and with a Pt/Ir coated tip (EFM tip, Nanoworld, Switzerland), resonance frequency of 75 kHz and a constant force of 2.8 N m^{-1}. Surface composition was determined by X-ray Photoelectron (XPS) and Raman scattering spectroscopies. Raman spectra and spectral images were collected in a Witec Alpha 300 micro-Raman confocal microscope (532 nm excitation laser and 20x objective lens) on a glassy carbon substrate and XPS spectra were acquired in a Thermo Scientific K-alpha spectrometer (Al Kα gun source, 300 μm spot size, 50.0 eV pass energy, and 0.100 eV step size). For the XPS and GIXRD characterization, Cu_2O NCs were drop-casted onto FTO substrate and dried in vacuum prior to the analysis (in the case of the pristine sample). The post-electrolysis sample was first washed with MilliQ water and then dried (in vacuum) before characterization. X-ray patterns were acquired in a Bruker D8 Advance Eco diffractometer (Cu Kα, 8 keV).

Electrochemical measurements

The electrochemical measurements were conducted by a Metrohm Autolab PGSTAT302N electrochemical workstation in a 2-compartment (H-type). The anodic and cathodic compartments were separated by a Fuel Cell Store Fumasep FAB-PK-130 membrane. For the preparation of Cu_2O NCs working electrodes, 20 μL of the solution containing 2 mg mL^{-1} of the nanoparticles and 1% v/v of a Nafion™ 5 wt% solution was drop-casted on a 5 mm diameter (0.196 cm^2 area) glassy carbon disk. The reference electrode was the reversible hydrogen electrode (RHE) and a platinum mesh (1.0 x 1.0 x 0.01 cm) counter electrode. To avoid any interference by changes in the bulk electrolyte pH a solution of 15 mL of a 1.0 mol L^{-1} NaOH was used in both cell compartments. Both compartments were degassed with ultra-high purity argon. To perform the nitrate reduction study, the linear sweep voltammetry (LSV) and chronoamperometry (CA) experiments were conducted by adding into the cathode compartment 14 mmol L^{-1} of nitrate. The potentiostatic test was carried out at different potentials (−0.2 to −0.6 V *vs.* RHE) for 1 h under a mass transport controlled by a rotation rate of 400 rpm. Chronoamperometric stability test was conducted at −0.2 V vs. RHE in 5 consecutive 2-h electrolysis in NaOH 1.0 mol L^{-1} and $NaNO_3$ 14 mmol L^{-1}. At each electrolysis, the electrolyte was collected for product quantification and renewed. During the electrolyte exchange, no potential was applied and the electrode was minimally exposed to air. The electrochemical active surface area (ECSA) was determined using the double-layer capacitance (C_{dl}) (Figure S1), which was measured by cyclic voltammetry (CV) in the range of 20-100 mV s^{-1} in a non-faradaic potential region. The C_{dl} was obtained from the plot of current density against scan rate. Thus, the ECSA can be calculated according to the Equation S1:

$$ECSA = C_{dl} / C_s \quad (S1)$$

The specific capacitance (C_s) for a flat surface is generally in the range of 20-60 μF cm^{-2}.[2] In our work, we used a value of 40 μF cm^{-2}.

Determination of NH_3 and NO_2^-

The ultraviolet-visible (UV-vis) spectrophotometer was used to detect the ions in the electrolyte. NH_3 quantification was conducted using the indophenol blue method. 500 μL of the samples were in 2.5 mL of water. Then, 500 μL of an alkaline sodium salicylate solution (0.3 mol L^{-1} NaOH and 0.4 mol L^{-1} sodium salicylate), 50 μL of 1% sodium nitroprusside (1 % wt), and 50 μL of NaClO were added. The sample spectra were taken from 500 to 800 nm and the maximum absorbance peak was identified at 657 nm.

Nitrite was quantified by the Griess method. In detail, 500 μL of the sample was diluted with 2.5 mL of water and 1 mL of a chromogenic agent (Griess reagent) composed of 0.1 g of *N*-(1-naphthyl) ethylenediamine hydrochloride, 1.0 g of sulfanilamide, 2.94 mL of H_2PO_4) in 50 mL of was added. For nitrite detection, the UV-vis spectra were acquired in the range of 400 to 700 nm, and the wavelength at 540 nm was chosen to determine the sample concentration.

Calculation of faradaic efficiency and the yield rate

The Faradaic efficiency was measured by the charge consumed for produced ammonia and nitrite according to Equation S2:

$$FE = \frac{n \times F \times c \times V}{i \times t} \cdot 100\% \qquad \text{(S2)}$$

The yield of NH_3 was calculated using the Equation S3:

$$Yield = \frac{c \times V}{t \times ECSA} \cdot 100\% \qquad \text{(S3)}$$

Where *n* is the number of electrons transferred (8 for NH_3 and 2 for NO_2^-), *F* is the Faraday constant (96500 C mol^{-1}), *c* is the concentration in mol L^{-1}, *V* is the catholyte volume (0.015 L), *i* is the total current, *t* is the electrolysis time (h), and *ECSA* is the electrochemical surface area (cm^2).

In situ Fourier Transform Infrared Spectroscopy

In situ Fourier Transform Infrared Spectroscopy (FTIR) experiments were carried out in a custom-made spectro-electrochemical cell (SEC) assembled on top of a CaF_2 window lined up on the top of a specular reflection accessory (Pike Technologies, model VeeMax II). The instrument used was a Shimadzu IR prestige-21 spectrometer equipped with a mercury-cadmium-telluride (MCT) detector refrigerated with liquid nitrogen. Platinum wire was used as a counter electrode and a reversible hydrogen electrode (RHE) as the reference. The working electrode (WE) was Cu_2O NCs deposited on a glassy carbon disk as described in the previous section (d = 5 mm). The WE was assembled into the cell and pressed against the CaF_2 window to create a thin layer. The spectra were collected during chronoamperometry measurements in 1.0 mol L^{-1} NaOH and 20 mmol L^{-1} $NaNO_3$ from 0.3 to −0.9 V *vs.* RHE with 100 mV intervals. The FTIR

spectra were obtained by external reflection from an average of 128 scans with a resolution of 4 cm^{-1} at each potential.

^{1}H Nuclear Magnetic Resonance

$^{14}NH_4^+$ and $^{15}NH_4^+$ were quantified by ^{1}H Nuclear Magnetic Resonance (NMR). After 1 h longer electrolysis at −0.4 V *vs*. RHE, an aliquot of the electrolyte was collected and acidified to pH = 3 to ensure the conversion of all NH_3 to NH_4^+. Then, 550 μL was transferred to a 5 mm NMR tube, and 100 μL D_2O was added to adjust the lock of the spectrometer. The ^{1}H NMR measurements were performed at room temperature on a Bruker AVANCE III NMR spectrometer, operating at 11.7 Tesla, observing ^{1}H nuclei at 500.13 MHz. The instrument was equipped with a direct detection probe. The ^{15}N isotope-labeling nitrate reduction experiments were conducted to validate that ammonia formation is exclusively fueled by the nitrate source. These experiments were analogous to the ^{1}H NMR procedure mentioned earlier, with the exception being the utilization of $Na^{15}NO_3$ (98 atom% ^{15}N) as the nitrate source. The spectra of ^{1}H NMR were acquired using pulse sequence zgesgp (Bruker library) using the following parameters: 2s relaxation delay (d1), 4 dummy scans (ds), 1 k transients (ns), 4.089 s acquisition time, 64 k data points distributed over 8012 Hz spectral width. The spectra were processed by the application of an exponential multiplication free induction decay (FID) with a line broadening factor of 0.3 Hz, followed by Fourier transform with zero filling by a factor of 2.

In situ Raman Spectroscopy

In situ Raman spectroscopy was performed in a Renishaw InVia microscope, equiped with a 633 nm laser. The spectro-electrochemical cell was assembled on top of a quartz window, in a three-electrode configuration cell: Cu_2O NCs ink (40μL) drop-casted on Au (0.385 cm^2) as the working electrode, a platinum wire as a counter electrode, a leakless Ag/AgCl as a reference electrode. The spectrums were collected during chronoamperometry measurements in a 1.0 mol L^{-1} NaOH electrolyte with and without 0.1 mol L^{-1} $NaNO_3$ from 0.8 to −0.4 V *vs*. RHE with 100 mV steps. *In situ* Raman spectra were obtained by the accumulation of 16 scans, with 100% laser power, and 1 s exposure time.

Online Differential Electrochemical Mass Spectrometry

Online Differential Electrochemical Mass Spectrometry (DEMS) experiments were conducted by coupling chronoamperometry (CA) measurements to the mass spectrometer to track possible gaseous or

volatile species by their ionic currents of mass/charge (m/z) ratios generated along the electrochemical nitrate reduction in a potential range from −0.3 to 0.8 V *vs*. RHE. The possible ionic species monitored were hydrogen (H_2), ammonia (NH_3), nitrogen (N_2), Diazenylium (N_2H^+), nitric oxide (NO), and hydroxylamine (NH_2OH) at m/z = 2, 17, 28, 29, 30, and 33, respectively. The working electrode was 100 μL of the Cu_2O NCs ink drop casted on a carbon cloth (thickness of 410 μm from Fuel Cell Store) and a PTFE membrane (Gore-Tex, 0.02 μm pore size and 50 μm thickness) underneath, attached to a holder (PTFE), which was screwed to the stainless-steel flange and placed inside a custom-made electrochemical cell. The exposed area of the working electrode to the solution was 0.38 cm^2 in all experiments. A platinum mesh and a reversible hydrogen electrode (RHE) were used as counter and reference electrodes, respectively. The experiments were conducted under Ar-saturated solutions kept under magnetic stirring (700 rpm). The electrolyte was 1.0 mol L^{-1} NaOH and 140 mmol L^{-1} of $NaNO_3$. Therefore, all reported ionic current values are relative and refer to changes from the initial baseline levels observed during the applied potential.

In situ X-ray Fluorescence, Absorption and Spectromicroscopy

Synchrotron experiments were carried out at beamline Carnaúba/Sirius, at Tarumã station, using a beam size of 200 nm x 500 nm (nanoprobe) with an estimated flux of about 109 photons/second on the sample. X-ray fluorescence mapping (nano-XRF) was carried out in continuous scan mode (called flyscan) over 50 μm x 50 μm with a step size of 500 nm (pixel size) by scanning the sample about the beam. Punctual in situ X-ray absorption spectroscopy (XAS) experiments were performed using a four-bounce Si(111) monochromator with energy resolution of 10^{-4} at a step of 0.5 eV. The punctual spectra and XANES maps were collected during chronoamperometry measurements with electrolyte containing 1.0 mol L^{-1} NaOH and 0.1 mol L^{-1} $NaNO_3$ from −0.4 to −1.0 V *vs*. RHE (at every 200 mV) using EC301 potentiostat from Stanford Research Systems. For each measurement, the potential was applied for 10 minutes before the spectra acquisition.

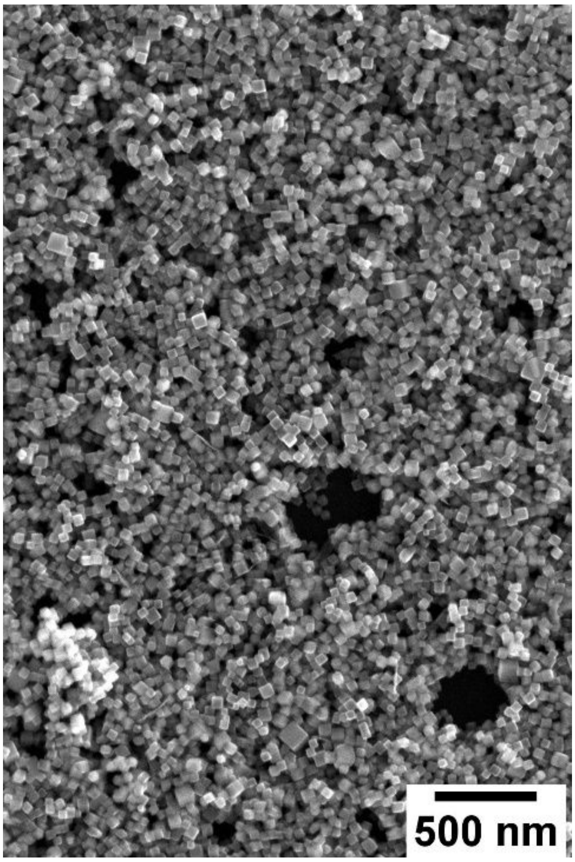

Figure S1. SEM image used for particle size determination.

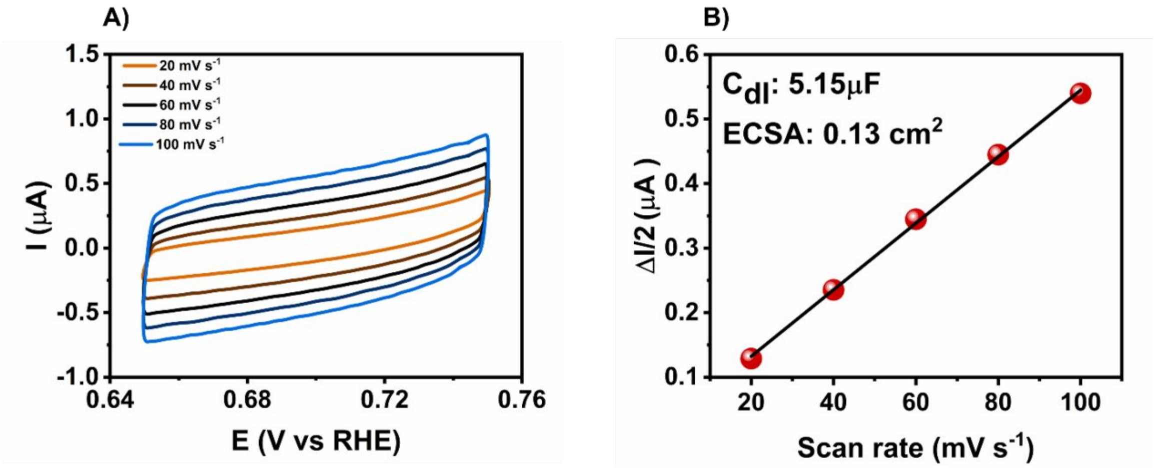

Figure S2. Electrochemically active surface area (ECSA) measurements. (A) Cyclic voltammograms of Cu_2O NCs at different scan rates (20 to 100 mV s^{-1}) in 1.0 mol L^{-1} NaOH electrolyte. (B) linear relationship (black line) for the modulus of the average of double-layer current and the scan rate.

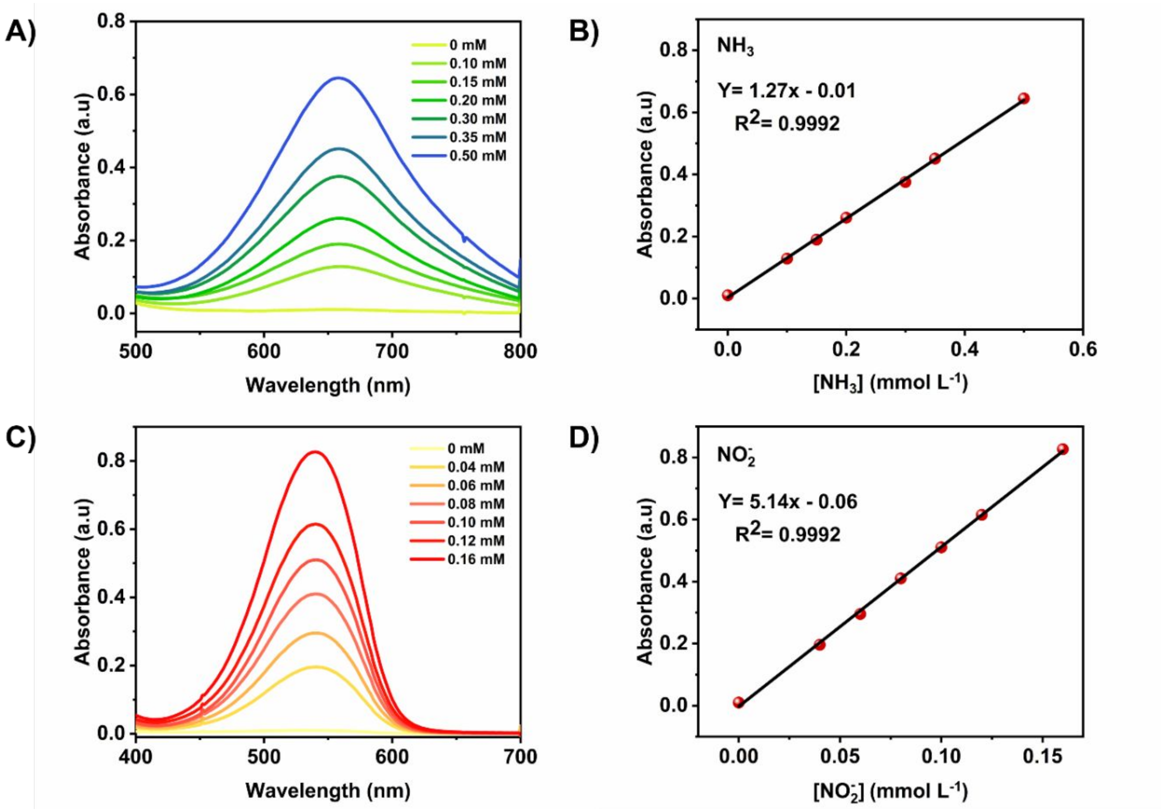

Figure S3. UV-vis spectra of A) NH_3 and (C) NO_2^- quantifications. Linear relationship between the maximum absorbance wavelength and species concentration for B) NH_3 at 657 nm and D) NO_2^- at 540 nm.

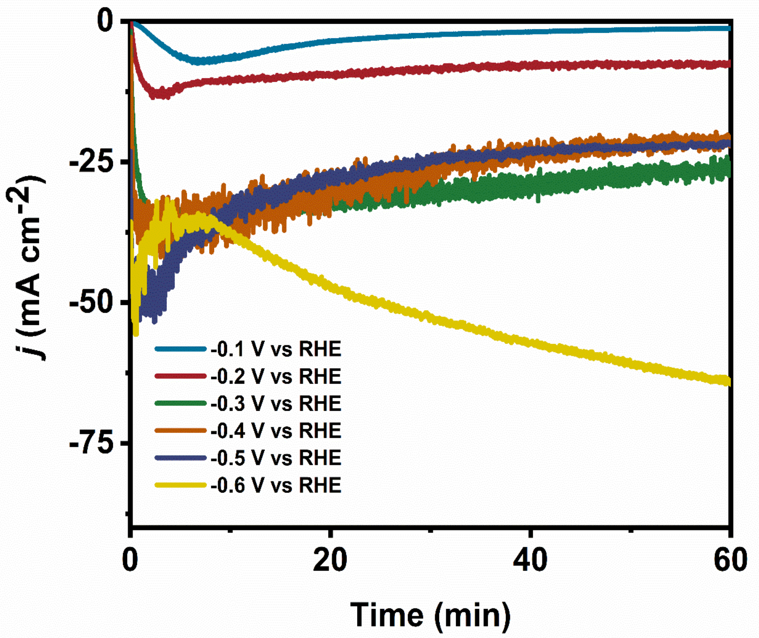

Figure S4. Chronoamperometry curves of Cu_2O NCs at different applied potentials from −0.1 V to −0.6 V *vs*. RHE in 1.0 mol L^{-1} NaOH with 14 mmol L^{-1} of NO_3^- under magnetic stirring at 400 rpm.

Table S1. Comparison of catalytic performance of Cu_2O NCs with others reported non-noble-metal catalysts for nitrate electroreduction to ammonia.

Catalyst	Electrolyte	Performance	Refs.
**Cu_2O NCs**	**1 mol L^{-1} NaOH + 14 mmol L^{-1} $NaNO_3$**	**FE_{NH3}(%) = 94 at −0.3 V *vs.* RHE**<br>**Y_{NH3} = 0.149 mmol h^{-1} cm^{-2}**	**This work**
Cu_2O (111)	1 mol L^{-1} NaOH + 0.1 mol L^{-1} $NaNO_3$	FE_{NH3}(%) = 76 at −0.30 V *vs.* RHE	[2]
Cu_2O (100)	1 mol L^{-1} NaOH + 0.1 mol L^{-1} $NaNO_3$	FE_{NH3}(%) = 68 at −0.30 V *vs.* RHE	[2]
Cu/Cu_2O NWAs	0.5 mol L^{-1} Na_2SO_4 + 14.3 mmol L^{-1} $NaNO_3$	FE_{NH3}(%) = 95.8 at −0.85 V *vs.* RHE<br>Y_{NH3} = 0.24 mmol h^{-1} cm^{-2}	[3]
TiO_{2-x}	0.5 mol L^{-1} Na_2SO_4 + 3.57 mmol L^{-1} $NaNO_3$	FE_{NH3}(%) = 85.0 at −0.95 V *vs.* RHE<br>Y_{NH3} = 0.045 mmol h^{-1} mg_{cat}^{-1}	[4]
Cu_2O-Cu/Ti	1 mol L^{-1} KOH +<br>40 mmol L^{-1} KNO_3	FE_{NH3}(%) = 92.0 at −0.5 V *vs.* RHE<br>Y_{NH3} = 0.28 mmol h^{-1} cm^{-2}	[5]
Cu foil	1 mol L^{-1} KOH +<br>100 mmol L^{-1} KNO_3	FE_{NH3}(%) = 92 at −0.15 V *vs.* RHE<br>Y_{NH3} = 1.1 mmol h^{-1} cm^{-2}	[6]
$_{V}Co$-Co_3O_4/CC	0.1 mol L^{-1} NaOH +<br>100 mmol L^{-1} $NaNO_3$	FE_{NH3}(%) = 97.2 at −0.6 V *vs.* RHE<br>Y_{NH3} = 0.51 mmol h^{-1} cm^{-2}	[7]
Fe_3O_4@TiO_2/TP	0.1 mol L-1 PBS +<br>100 mmol L-1 NaNO3	FE_{NH3}(%) = 88.4 at −0.9 V *vs.* RHE<br>Y_{NH3} = 0.73 mmol h^{-1} cm^{-2}	[8]
OV-rich MnO2−x	0.5 mol L^{-1} Na_2SO_4 +<br>100 mmol L^{-1} $NaNO_3$	FE_{NH3}(%) = 92.4 at −1.0 V *vs.* RHE<br>Y_{NH3} = 0.19 mmol h^{-1} cm^{-2}	[9]
Cu nanodisks	1 mol L^{-1} KOH +<br>10 mmol L^{-1} KNO_3	FE_{NH3}(%) = 81.1 at −0.5 V *vs.* RHE<br>Y_{NH3} = 0.12 mmol h^{-1} mg_{cat}^{-1}	[10]
Mo-SnO_{2-x}	0.5 mol L^{-1} Na_2SO_4 +<br>100 mmol L^{-1} $NaNO_3$	FE_{NH3}(%) = 95.5 at −0.7 V *vs.* RHE<br>Y_{NH3} = 0.31 mmol h^{-1} cm^{-2}	[11]
Fe_2O_3 NRs/CC	0.5 mol L^{-1} Na_2SO_4 +<br>100 mmol L^{-1} $NaNO_3$	FE_{NH3}(%) = 69.7 at −0.9 V *vs.* RHE<br>Y_{NH3} = 0.33 mmol h^{-1} cm^{-2}	[12]

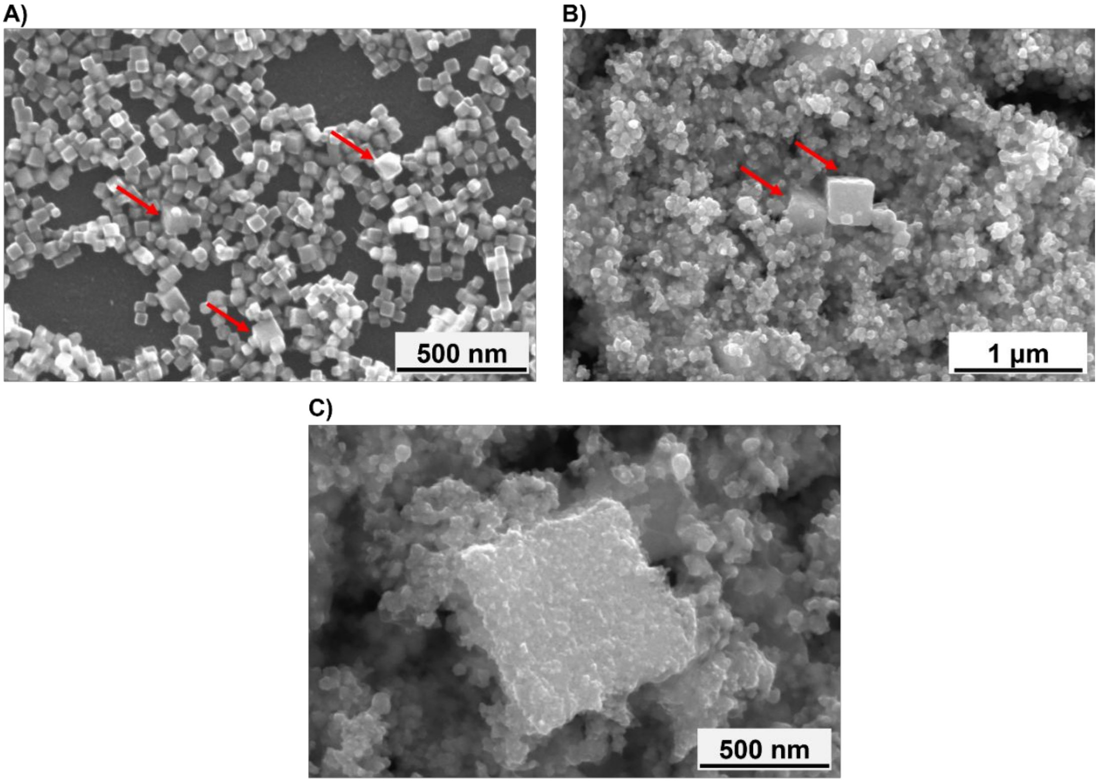

Figure S5. SEM images of the electrocatalyst A) before and B-C) after 1-hour of NO_3RR at −0.3 V *vs*. RHE in 1.0 mol L^{-1} NaOH with 14 mmol L^{-1} of $NaNO_3$ at medium and high magnification, respectively. The red arrows indicate larger nanocubes particles domains.

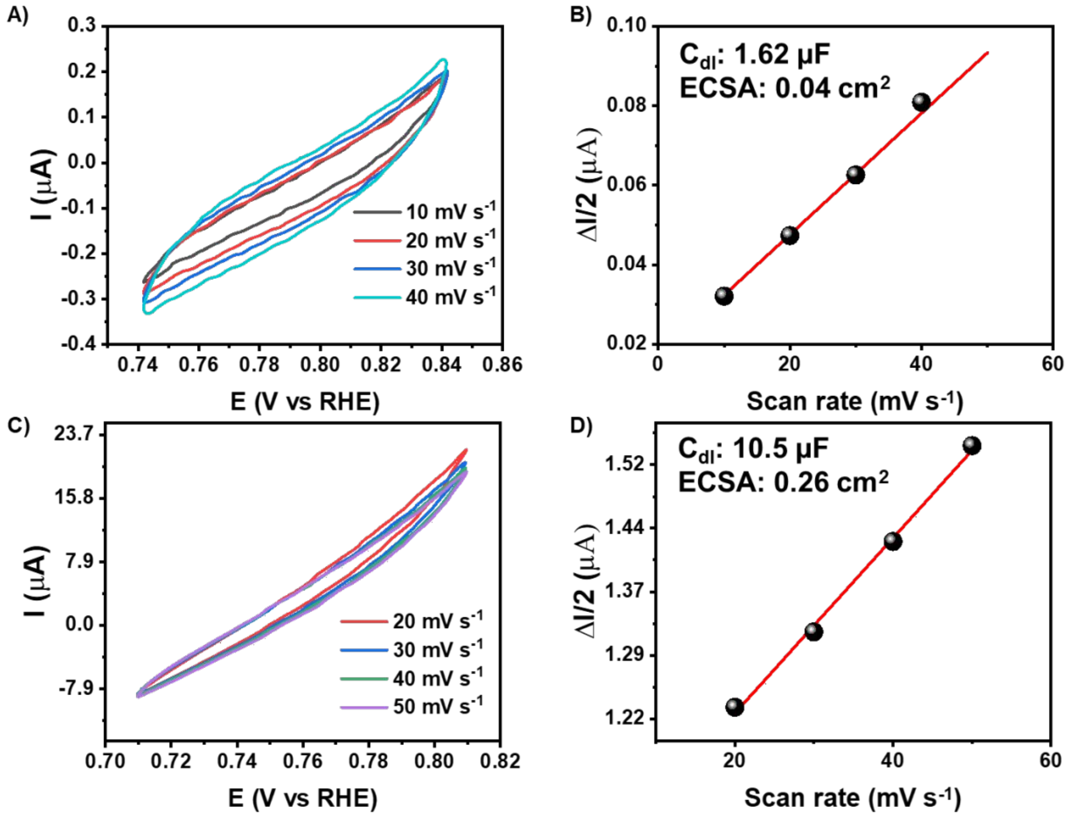

Figure S6. Electrochemically active surface area (ECSA) measurements of Cu_2O electrode before and after 1-h electrolysis (-0.3 V *vs.* RHE in 1.0 mol L^{-1} NaOH and 14 mmol L^{-1} $NaNO_3$). Cyclic voltammograms of Cu_2O NCs and linear relationship (black line) for the modulus of the average of double-layer current and the scan rate A-B) before applying cathodic potential and C-D) after. Cyclic voltammograms were obtained in 1.0 mol L^{-1} NaOH.

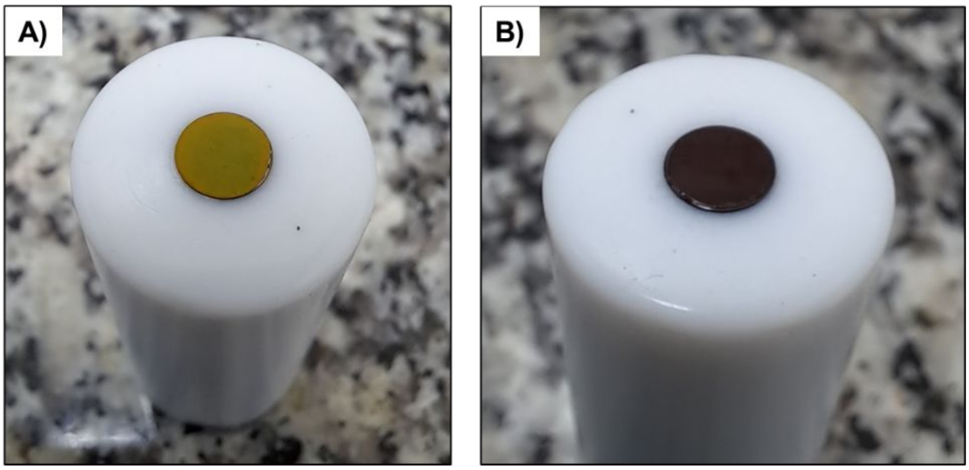

Figure S7. Photographs of the Cu_2O NCs modified glassy carbon electrode A) before and B) after 1-hour chronoamperometry at −0.3 V *vs*. RHE in NaOH 1.0 mol L^{-1} and $NaNO_3$ 14 mmol L^{-1}.

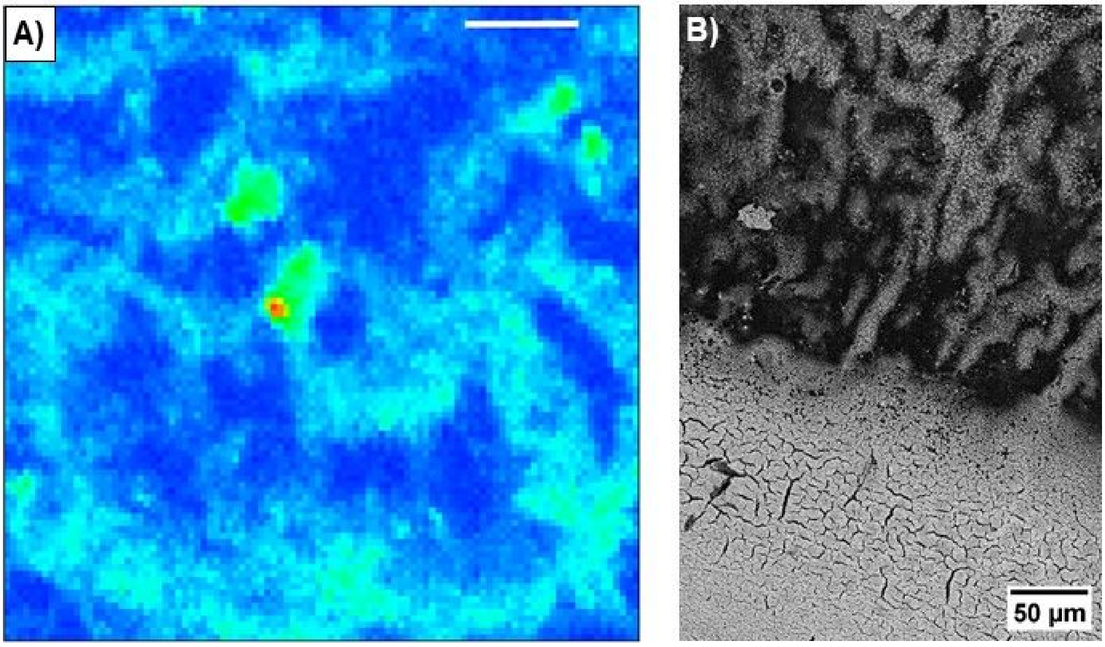

Figure S8. Post-electrolysis characterization of Cu_2O NCs by A) XRF mapping of a 50 μm x 50 μm analyzed area (500 nm/pixel; scale bar denotes 10 μm). Redder regions represent higher Cu concentration; and B) low-magnification SEM micrograph.

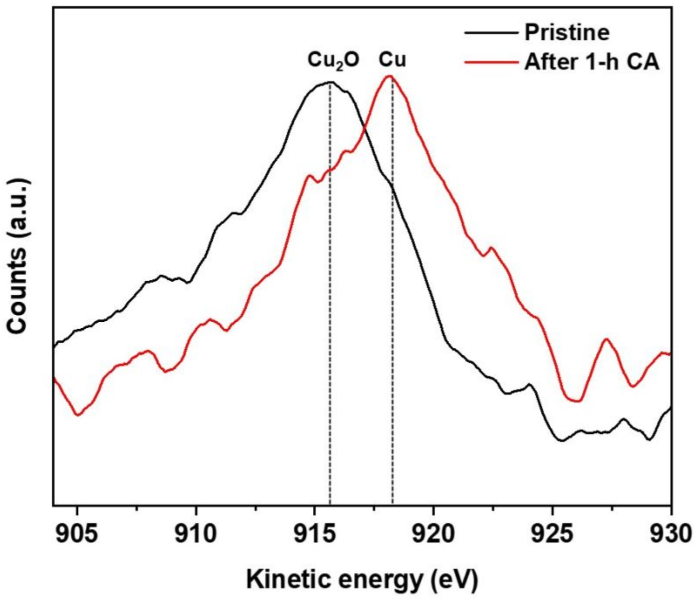

Figure S9. Cu LMM spectra of pristine and post-electrolysis Cu_2O NCs (1-h at –0.3 V *vs.* RHE in NaOH 1.0 mol L^{-1} and $NaNO_3$ 14 mmol L^{-1}).

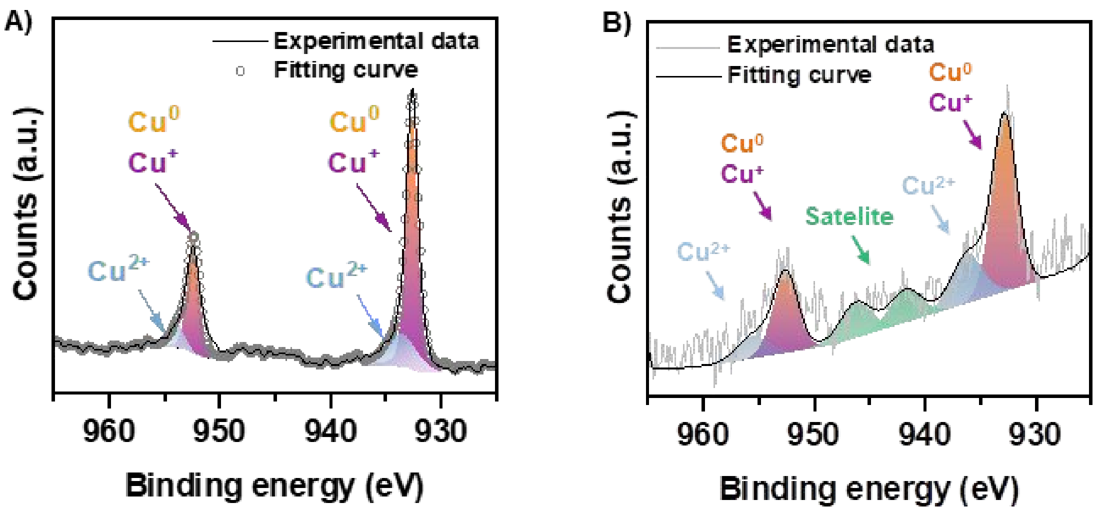

Figure S10. High-resolution Cu 2p XPS spectra of two post-electrolysis Cu_2O NCs (1-h at −0.3 V vs. RHE in 1.0 mol L^{-1} NaOH and 14 mmol L^{-1} $NaNO_3$). A) Experiment 1 and B) 2.

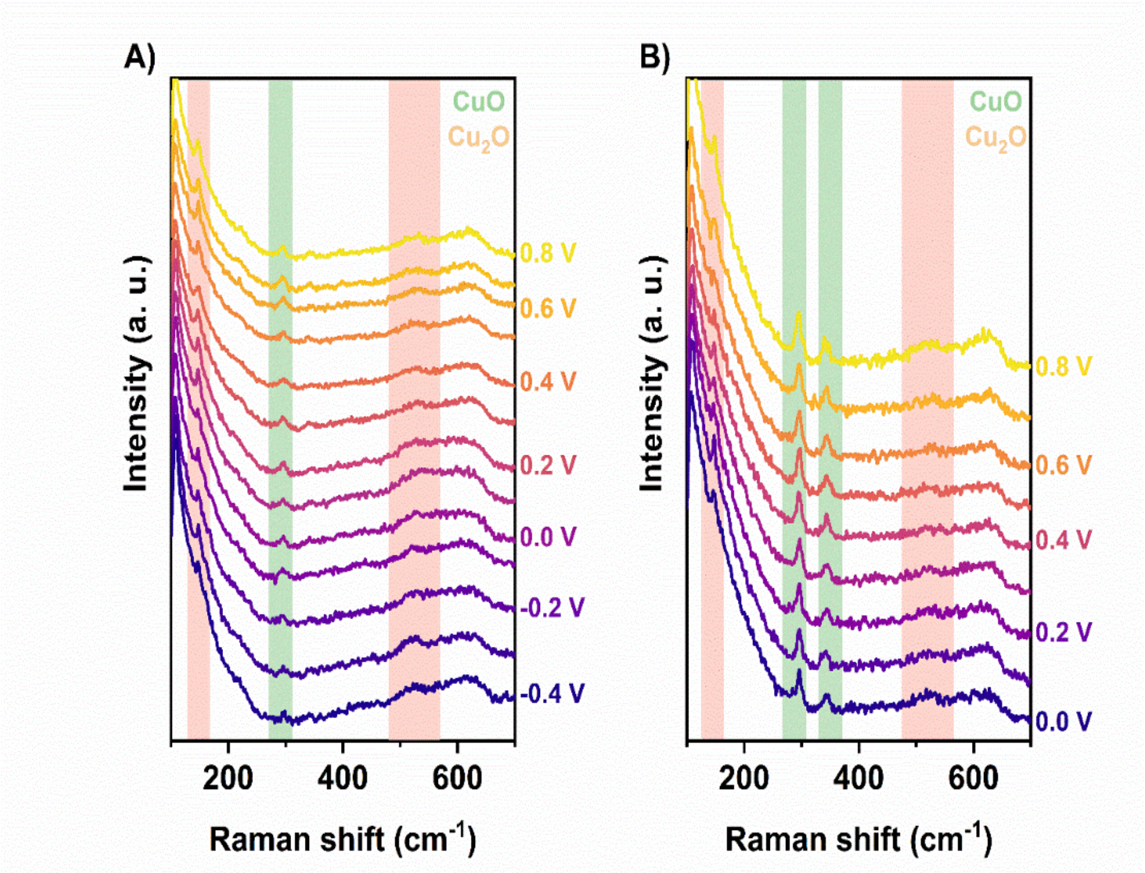

Figure S11. *In situ* Raman spectra A) without (potential range: 0.8 to -0.4 V *vs.* RHE) and B) with 0.1 mol L^{-1} of $NaNO_3$ (potential range: 0.8 to 0 V *vs.* RHE) in 1.0 mol L^{-1} NaOH electrolyte. Pink marks highlight the peaks related to Cu_2O (145, 520, 630 cm^{-1}) and green ones highlight peaks related to CuO (295, 340 cm^{-1}).

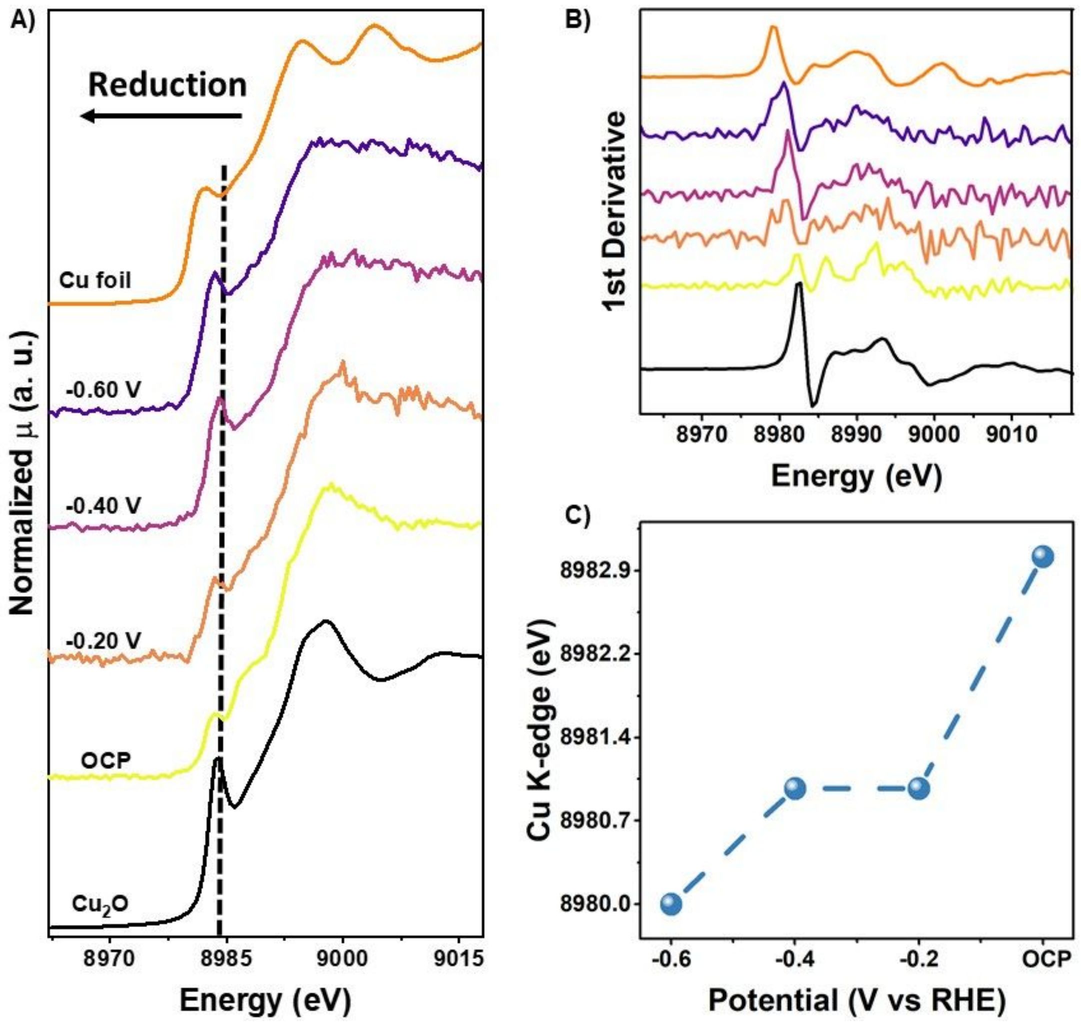

Figure S12. A) Normalized *in situ* XANES spectra of Cu_2O NCs at different applied potentials in 1.0 mol L^{-1} NaOH and 14 mmol L^{-1}, and the spectra of the standards Cu and Cu_2O; B) the corresponding first derivative curves of the *in situ* XANES spectra; and C) dependence of the Cu K-edge energy as a function of the electrochemical potential.

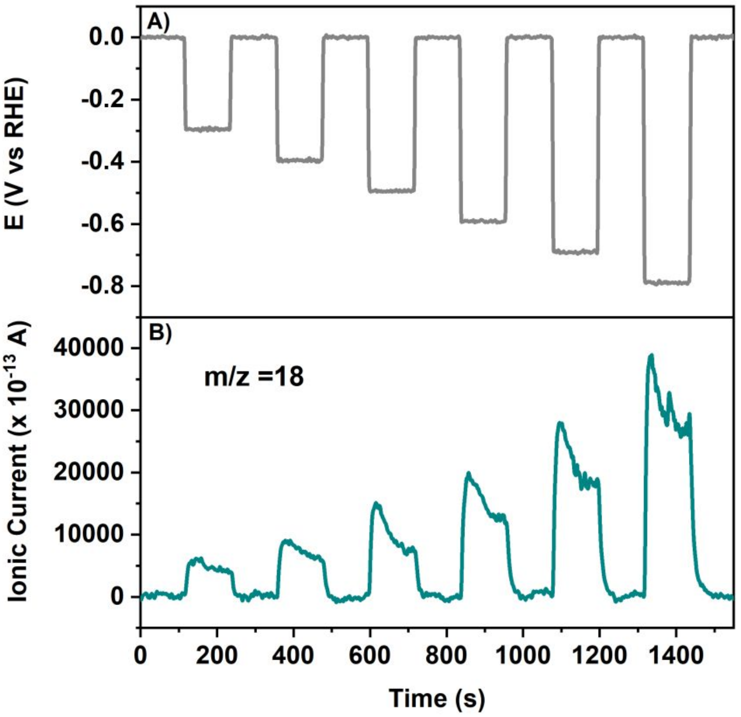

Figure S13. Online DEMS of Cu_2O NCs recorded in 0.1 mol L^{-1} NaOH + 14 mmol L^{-1} $NaNO_3$ electrolyte. A) Applied potential *vs*. time. B) Ionic current *vs*. time of the fragment m/z = 18 of H_2O molecules.

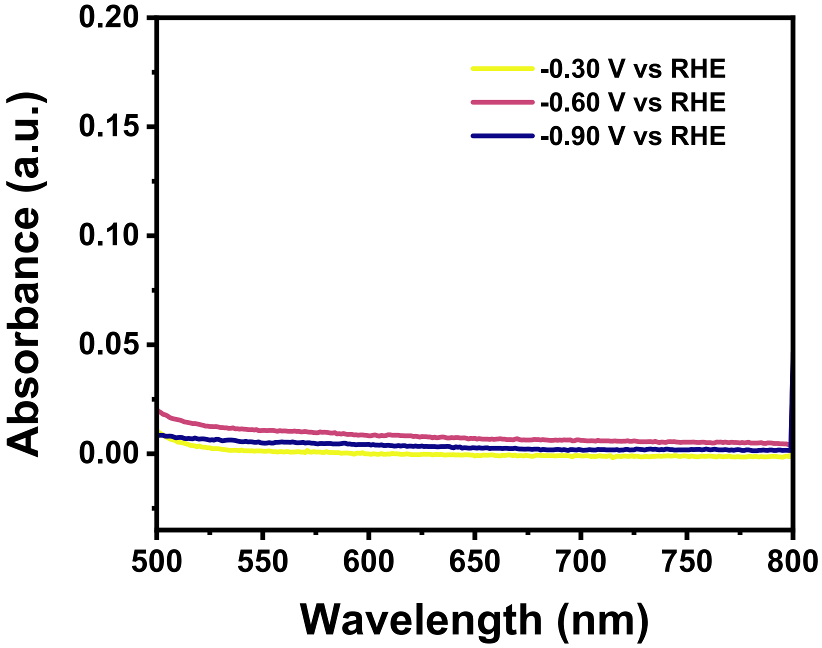

Figure S14. UV-vis spectrum of ammonia quantification by chronoamperometry at −0.30 V, −0.60 V and −0.90 V *vs*. RHE in 1.0 mol L^{-1} NaOH and 0.20 mol L^{-1} N_2H_4.

REFERENCES

(1) A. Herzog, A. Bergmann, H. S. Jeon, J. Timoshenko, S. Kühl, C. Rettenmaier, M. Lopez Luna, F. T. Haase, B. Roldan Cuenya, *Angewandte Chemie International Edition* **2021**, *60*, 7426-7435.

(2) D. Anastasiadou, Y. van Beek, W. Chen, T. Wissink, A. Parastaev, E. J. M. Hensen, M. Costa Figueiredo, *ChemCatChem* **2023**, *15*, e202201503.

(3) Y. Wang, W. Zhou, R. Jia, Y. Yu, B. Zhang, *Angewandte Chemie International Edition* **2020**, *59*, 5350-5354.

(4) R. Jia, Y. Wang, C. Wang, Y. Ling, Y. Yu, B. Zhang, *ACS Catalysis* **2020**, *10*, 3533-3540.

(5) M. E. Chavez, M. Biset-Peiró, S. Murcia-López, J. R. Morante, *ACS Sustainable Chemistry & Engineering* **2023**, *11*, 3633-3643.

(6) J. Yuan, Z. Xing, Y. Tang, C. Liu, *ACS Applied Materials & Interfaces* **2021**, *13*, 52469-52478.

(7) Z. Deng, C. Ma, Z. Li, Y. Luo, L. Zhang, S. Sun, Q. Liu, J. Du, Q. Lu, B. Zheng, X. Sun, *ACS Applied Materials & Interfaces* **2022**, *14*, 46595-46602.

(8) X. He, J. Li, R. Li, D. Zhao, L. Zhang, X. Ji, X. Fan, J. Chen, Y. Wang, Y. Luo, D. Zheng, L. Xie, S. Sun, Z. Cai, Q. Liu, K. Ma, X. Sun, *Inorganic Chemistry* **2023**, *62*, 25-29.

(9) G. Wang, P. Shen, Y. Luo, X. Li, X. Li, K. Chu, *Dalton Transactions* **2022**, *51*, 9206-9212.

(10) K. Wu, C. Sun, Z. Wang, Q. Song, X. Bai, X. Yu, Q. Li, Z. Wang, H. Zhang, J. Zhang, X. Tong, Y. Liang, A. Khosla, Z. Zhao, *ACS Materials Letters* **2022**, *4*, 650-656.

(11) G. Zhang, N. Zhang, K. Chen, X. Zhao, K. Chu, *Journal of Colloid and Interface Science* **2023**, *649*, 724-730.

(12) T. Li, C. Tang, H. Guo, H. Wu, C. Duan, H. Wang, F. Zhang, Y. Cao, G. Yang, Y. Zhou, *ACS Applied Materials & Interfaces* **2022**, *14*, 49765-49773.
